# Insights into the local structure and optical performance of SrY_2_O_4_:Eu^3+^ red phosphors through Judd–Ofelt analysis

**DOI:** 10.1039/d6ra05000j

**Published:** 2026-09-01

**Authors:** Vu Thi Kim Lien, Le Thi Phuong, Vu Van Thu, Nguyen Dac Dien, Le Thi Oanh, Do Thi Lan Chi, Pham Thi Lien, Chu Anh Tuan, Pham Van Tuan, Hoang Gia Chuc, Manh Trung Tran, Le Tien Ha

**Affiliations:** a Faculty of Natural Sciences, Duy Tan University Da Nang 550000 Vietnam; b Institute of Theoretical and Applied Research, Duy Tan University Hanoi 100000 Vietnam; c TNU - University of Sciences Thai Nguyen 250000 Vietnam; d Faculty of Occupational Safety and Health, Trade Union University Hanoi 10000 Vietnam thuvv@dhcd.edu.vn; e Institute of Materials Science, Vietnam Academy of Science and Technology 18 Hoang Quoc Viet, Nghia Do Hanoi Vietnam; f Viet Nam University of Traditional Medicine Hanoi 100000 Vietnam; g School of Materials Science and Engineering, Hanoi University of Science and Technology No. 1, Dai Co Viet Hanoi Vietnam; h Faculty of Materials Science and Engineering, Phenikaa School of Engineering, Phenikaa University Nguyen Trac, Duong Noi Hanoi Vietnam

## Abstract

In this study, Eu^3+^-doped SrY_2_O_4_ red-emitting phosphors were successfully synthesized *via* a conventional solid-state reaction route, and their structural, optical and luminescence properties were systematically investigated. X-ray diffraction analysis confirmed the formation of a single-phase orthorhombic SrY_2_O_4_ host, while microscopic and spectroscopic studies revealed high crystallinity and homogeneous incorporation of Eu^3+^ ions into the lattice. Increasing the Eu^3+^ concentration from 1 to 6 mol% induced a slight lattice expansion and reduced the optical band gap from 6.13 to 5.30 eV. Under near-ultraviolet and blue-light excitation, the phosphors exhibited intense red emission dominated by the ^5^D_0_ → ^7^F_J_ transition of Eu^3+^ ions, indicating the occupation of non-centrosymmetric lattice sites. Judd–Ofelt analysis was employed to evaluate the local environment and radiative emission properties of Eu^3+^ ions; the results indicate that Eu^3+^ ions occupy non-centrosymmetric sites with local asymmetry at *C*_s_ sites, with the *Ω*_2_ value increasing slightly from 2.623 × 10^−20^ to 2.795 × 10^−20^ cm^2^ as the Eu concentration rises from 1% to 5%. The optimum emission intensity was achieved at 5 mol% Eu^3+^, beyond which concentration quenching occurred through non-radiative energy transfer between neighboring activator ions. The phosphors demonstrated excellent thermal stability with an average activation energy of 0.242 eV, high color purity exceeding 85%, and an internal quantum efficiency of 67.74%. These results highlight the potential of SrY_2_O_4_:Eu^3+^ phosphors as efficient red-emitting components for warm white light-emitting diode (WLED) technologies.

## Abstract 2

In this study, Eu^3+^-doped SrY_2_O_4_ red-emitting phosphors were successfully synthesized *via* a conventional solid-state reaction route, and their structural, optical and luminescence properties were systematically investigated.

## Graphical abstract

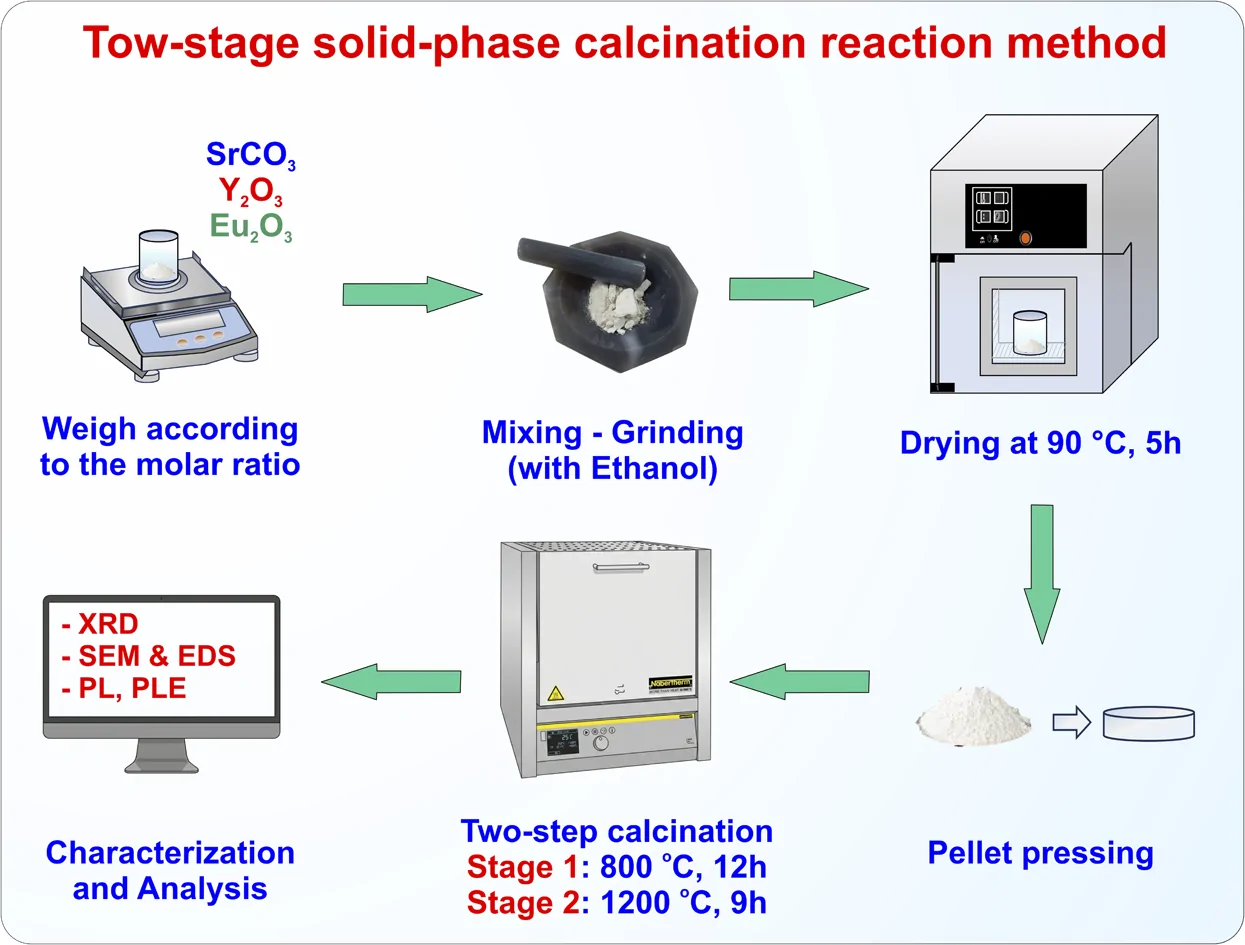

## 1. Introduction

White light-emitting diodes (WLEDs) have revolutionized solid-state lighting due to their high luminous efficiency, energy conservation, and long operational lifespan.^1–4^ Currently, the most common commercial configuration employs a blue InGaN chip combined with a yellow-emitting Y_3_Al_5_O_12_:Ce^3+^ (YAG:Ce) phosphor.^1–4^ While this “cold-white” solution is highly efficient, it suffers from a significant deficit in the red spectral region, resulting in a high correlated color temperature (CCT) and a poor color rendering index (CRI). These limitations render them unsuitable for high-quality indoor and residential lighting, which demands “warm-white” light—characterized by lower CCT and superior color fidelity.^5–8^ Achieving warm-white emission requires the integration of efficient red-emitting phosphors that can effectively fill the red spectral gap.

Rare-earth-activated oxide phosphors have emerged as promising candidates for this purpose, owing to their superior chemical stability, thermal robustness, and resistance to moisture compared to sulfide or nitride counterparts.^9–11^ Within the broad landscape of oxide hosts, materials such as Y_2_O_3_:Eu^3+^, Gd_2_O_3_:Eu^3+^, and complex ternary oxides like SrY_2_O_4_ have been extensively studied.^12,13^ Specifically, the SrY_2_O_4_ host is particularly intriguing due to its orthorhombic crystal structure, which contains two crystallographically distinct Y^3+^ sites with low local symmetry (*C*_s_).^14,15^ Such non-centrosymmetric environments are highly conducive to rare-earth incorporation, as they facilitate the relaxation of parity-selection rules governing 4f–4f transitions, thereby enhancing the radiative transition probabilities of Eu^3+^ ions.^10,15,16^

Despite the potential of rare-earth-doped oxide phosphors, the optimization of these materials for high-performance LEDs remains a significant challenge. While various synthesis routes, including sol–gel,^17^ electrospinning,^10^ and solid-state reactions,^18^ have been employed for SrY_2_O_4_:Eu^3+^, previous literature has primarily focused on empirical synthesis optimization and basic concentration-dependent luminescence behaviors.^10,15,19–23^ Notably, there remains a critical lack of fundamental understanding regarding the interplay between Eu^3+^ doping and the resulting modifications to the host lattice such as electronic band-gap evolution, precise radiative transition probabilities, and local symmetry dynamics. Existing studies often treat structural and optical features as isolated observations rather than correlated phenomena.

To address these limitations, this work provides a comprehensive, mechanistically driven investigation of Eu^3+^-doped SrY_2_O_4_ phosphors synthesized *via* a conventional solid-state reaction. By integrating structural characterization, Judd–Ofelt intensity analysis, and temperature-dependent photoluminescence studies, we elucidate the fundamental structure–property relationships governing the luminescent performance. This systematic approach aims to move beyond empirical reporting, offering deeper insights into the local coordination environment of the Eu^3+^ ions and the mechanisms limiting thermal stability, thereby positioning SrY_2_O_4_:Eu^3+^ as a viable candidate for warm-white LED applications.

## 2. Materials and methods

### 2.1. Materials

A series of SrY_2_O_4_:*x*% Eu^3+^ (*x* = 1–6) samples were synthesized *via* a conventional solid-state reaction method. High-purity SrCO_3_ (purity >99.9%), yttrium oxide (Y_2_O_3_, purity >99.9%), and europium(iii) (Eu_2_O_3_, purity >99.9%) were used as starting materials without further purification. The appropriate stoichiometric amounts of the precursors were thoroughly mixed and ground in an agate mortar using ethanol as a dispersing medium. The resulting slurry was dried at 90 °C for 5 h, pressed into pellets, and pre-calcined at 800 °C for 12 h in air. After cooling, the pre-calcined products were reground to ensure compositional homogeneity and subsequently sintered at 1200 °C for 9 h in air with a heating rate of 300 °C h^−1^. The furnace was then allowed to cool naturally to room temperature. The obtained products were ground into fine powders for subsequent structural and optical characterization.

### 2.2. Characterization

**Fig. 1:**
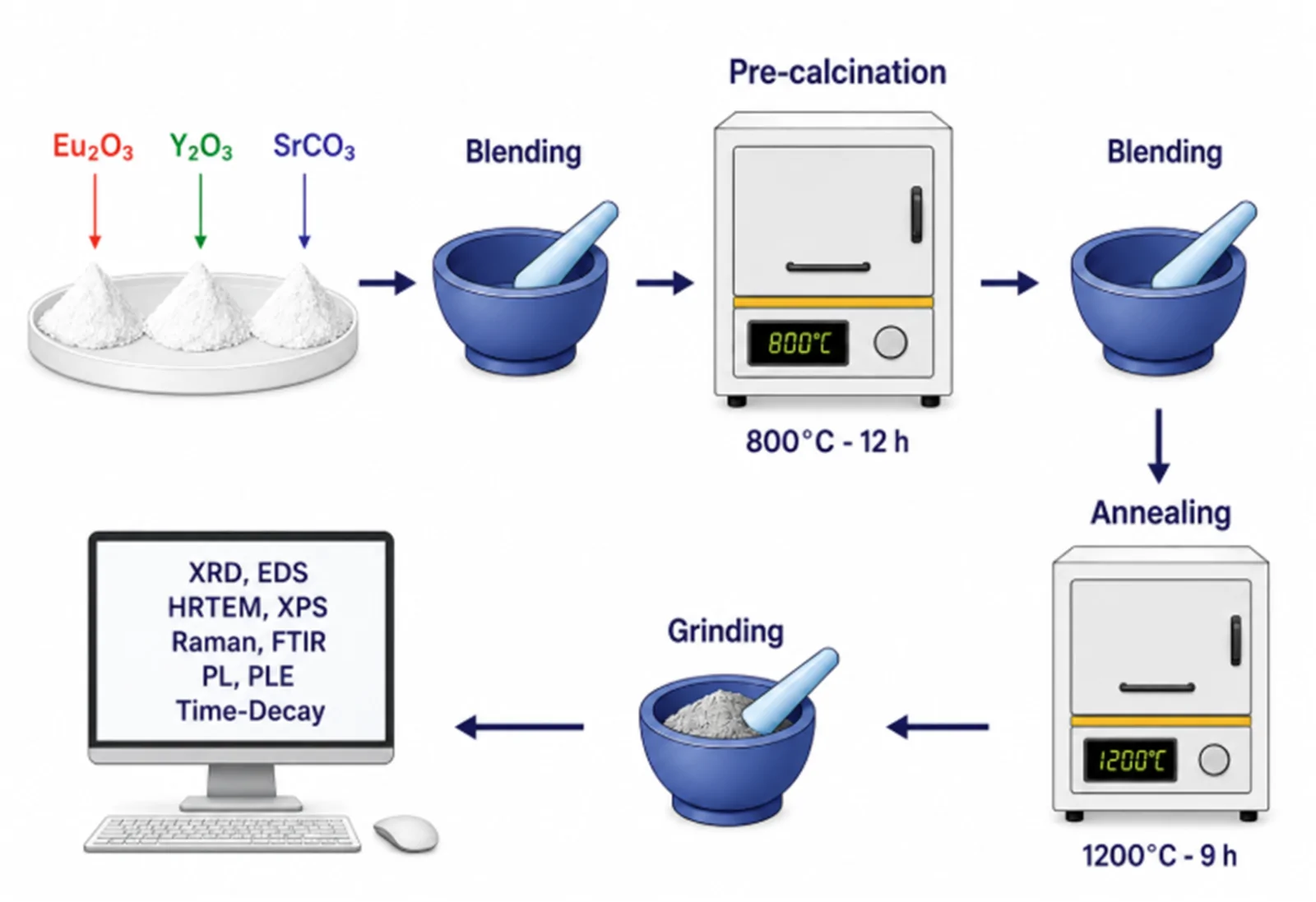
Synthesis process of red-emitting SrY_2_O_4_:Eu^3+^ luminescent material *via* the solid-state reaction method and methods for analyzing material properties.

The phase composition and crystal structure of the samples were examined by powder X-ray diffraction (XRD) using a D2 Advance diffractometer (Bruker, Germany) over a 2*θ* range of 20–80°. High-resolution transmission electron microscopy (HR-TEM) measurements were performed on a JEM2500SE microscope (JEOL, Japan) operated at 200 kV to investigate the microstructure and lattice fringes. Raman spectra recorded using an XploRa Plus spectrometer (Horiba, France) equipped with a 785 nm laser excitation source. Fourier-transform infrared (FT-IR) spectra were collected using a Spectrum Two spectrometer (PerkinElmer, USA) in the spectral range of 450–4000 cm^−1^. The morphology and elemental composition of the samples were characterized by field-emission scanning electron microscopy (FESEM) coupled with energy-dispersive X-ray spectroscopy (EDS) using a JSM-7600F microscope (JEOL, Japan). X-ray photoelectron spectroscopy (XPS) measurements were carried out on a Thermo Scientific spectrometer (USA) employing monochromatic Al Kα radiation. The binding-energy scale was calibrated using the adventitious carbon C 1s peak at 284.8 eV. Diffuse reflectance spectra were recorded and converted using the Kubelka–Munk function to evaluate the optical band-gap energy. Photoluminescence excitation (PLE) and emission (PL) spectra were recorded using a HORIBA NanoLog spectrofluorometer equipped with a 450 W xenon lamp. Both monochromators were operated with a slit setting of 1 and a 1200 grooves mm^−1^ grating blazed at 300 nm, while a 500 nm long-pass filter was used to suppress second-order diffraction. Emission spectra were detected using the T2 detector over 500–850 nm, with an integration time of 0.2 s per point and a step size of 1 nm. Luminescence decay curves were recorded using an FLS1000 spectrometer (Edinburgh Instruments, UK) equipped with a microsecond Xenon flashlamp (µF2), featuring a spectral range of 200–1000 nm and a pulse width of 1.0–2.0 µs. In this study, measurements were performed using a channel width of 4.0 µs. Temperature-dependent luminescence measurements were performed to evaluate the thermal stability of the phosphors. We have summarized the entire experimental process in Fig. 1.

## 3. Results and discussion

### 3.1. Structure and microstructure of materials

#### 3.1.1. XRD patterns

**Fig. 2:**
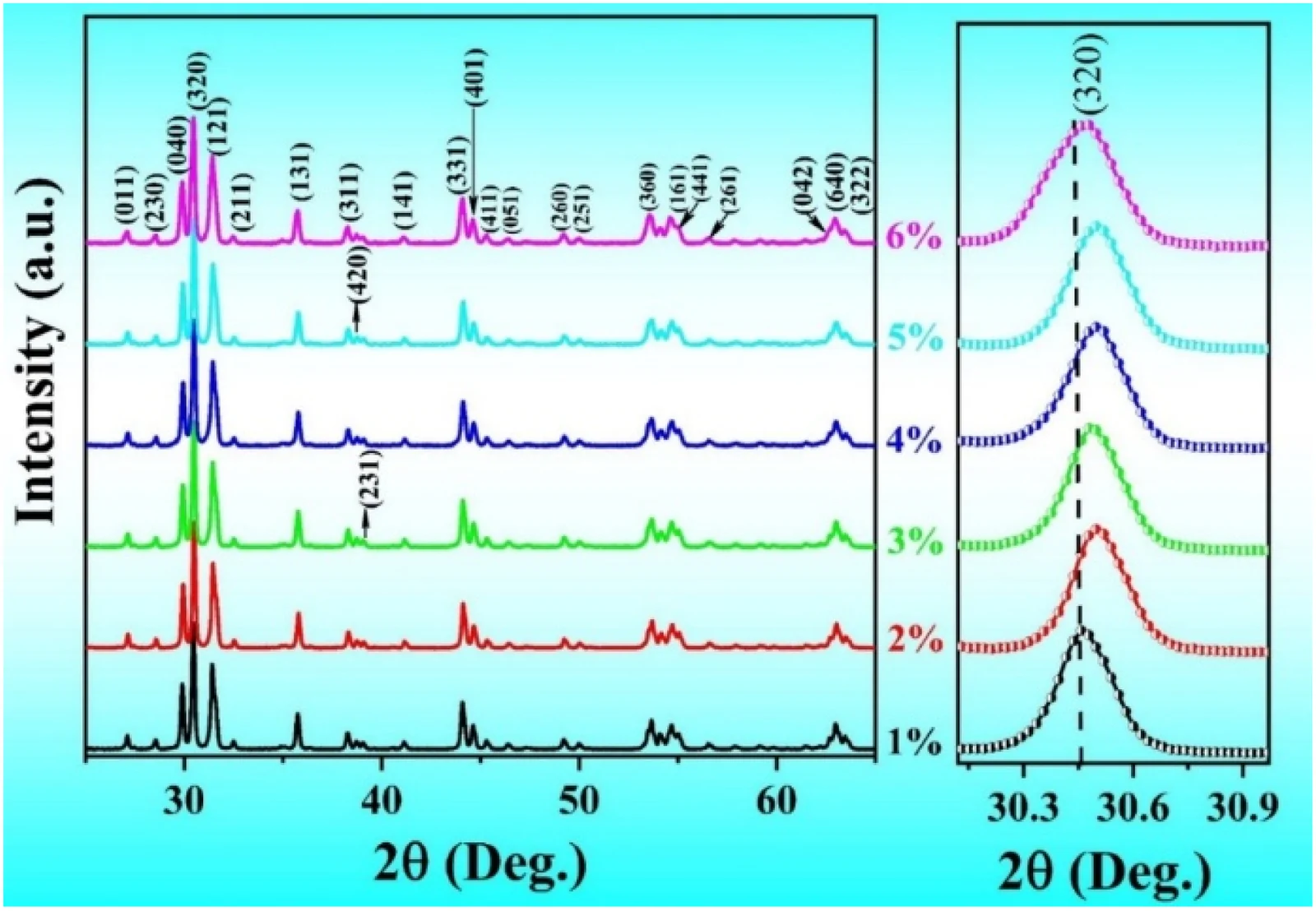
X-ray diffraction patterns of Eu-doped SrY_2_O_4_ samples at concentrations from 1 to 6%, calcined at 1200 °C in air.

The crystal structure of the of SrY_2_O_4_:*x*% Eu^3+^ (*x* = 1–6 mol%) phosphors was investigated by X-ray diffraction (XRD). Fig. 2 presents the diffraction patterns of the samples calcined at 1200 °C in air. All diffraction peaks can be indexed to the orthorhombic SrY_2_O_4_ phase with the *Pnam* space group, corresponding to the crystal planes (011), (230), (040), (320), (121), (211), (131), (311), and (322), located at 2*θ* values of approximately 27.11°, 28.62°, 29.92°, 30.48°, 31.41°, 32.44°, 35.76°, 38.28°, and 63.75°, respectively. The diffraction patterns are in good agreement with the standard PDF card No. 32-1272, confirming the formation of a single-phase SrY_2_O_4_ host lattice. Furthermore, the sharp diffraction peaks indicate the high crystallinity of the synthesized phosphors.^24^

No impurity phases associated with europium oxides or other secondary compounds were detected, indicating that the synthesized samples maintain a highly phase-pure structure without detectable phase segregation over the investigated concentration range. To confirm whether the Eu^3+^ ions were successfully incorporated into the SrY_2_O_4_ host lattice and to identify their preferential substitution sites, the ionic-radius difference (*D*_r_) between the host and dopant ions was evaluated using the following expression:^19^1
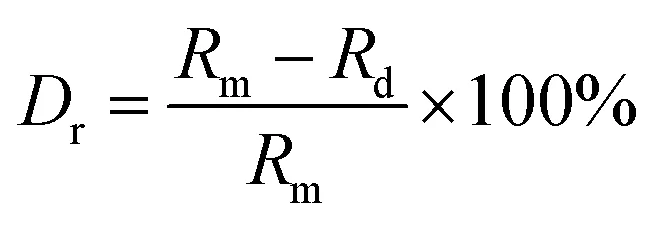
where *R*_m_ (CN) is the cation radius of the matrix, *R*_d_ (CN) is the radius of the impurity ion.

In the orthorhombic SrY_2_O_4_ structure, Sr^2+^ ions occupy two coordination environments with coordination numbers (CN) of 8 and 9 and ionic radii of 1.26 and 1.31 Å, respectively. In contrast, Y^3+^ ions occupy octahedral sites (CN = 6) with an ionic radius of approximately 0.90 Å. The ionic radius of Eu^3+^ is 0.947 Å for the same coordination number. The calculated D_r_ values for Eu^3+^ substitution at the Sr^2+^ (CN = 8), Sr^2+^ (CN = 9), and Y^3+^ sites are 24.84%, 27.71%, and 5.22%, respectively. Since the radius mismatch between Eu^3+^ and Y^3+^ is considerably smaller than that for Sr^2+^ sites, Eu^3+^ ions preferentially substitute for Y^3+^ ions in the SrY_2_O_4_ lattice. This substitution is also favored by the identical trivalent charge states of Eu^3+^ and Y^3+^, which minimizes charge-compensation effects.^19^

A magnified view of the (320) diffraction peak is shown at the inset of Fig. 2. As the Eu^3+^ concentration increases from 1 to 6 mol%, the diffraction peak exhibits a slight shift toward lower diffraction angles. According to Bragg's law, this behavior indicates an increase in interplanar spacing and a slight expansion of the crystal lattice. Such a shift is consistent with the substitution of smaller Y^3+^ ions (0.90 Å) by the slightly larger Eu^3+^ ions (0.947 Å), providing further evidence for the successful incorporation of Eu^3+^ into the host structure. Similar lattice expansion induced by rare-earth substitution has been reported for related oxide phosphor systems.

The average crystallite size was estimated from the (320) diffraction peak using the Scherrer equation:2
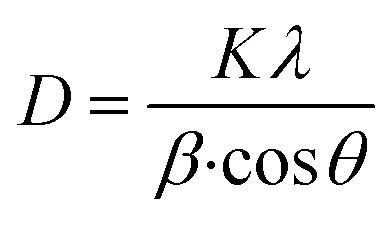
where *D* is the average crystallite size, *K* is the shape factor (0.89), *λ* = 1.540 is the X-ray wavelength, *β* is the full width at half maximum measured in radians, and *θ* is the Bragg angle. The calculated crystallite sizes are summarized in Table 1. The crystallite size decreases from 38.28 nm for the 1 mol% Eu^3+^ sample to 32.20 nm for the 6 mol% Eu^3+^ sample, accompanied by a gradual increase in FWHM. This result suggests that Eu^3+^ incorporation suppresses crystallite growth during the high-temperature calcination process. The reduction in crystallite size may be attributed to lattice distortion introduced by Eu^3+^ substitution, which hinders atomic diffusion and grain growth during annealing.

**Table 1:** Crystal size of SrY_2_O_4_:*x*% Eu^3+^ samples calcined at 1200 °C

Samples	2 *θ* (deg)	FWHM (deg)	Crystallite size in (320) direction (nm)
SrY_2_O_4_:1% Eu^3+^	30.5056	0.2127	**38.28**
SrY_2_O_4_:2% Eu^3+^	30.4915	0.2162	**37.66**
SrY_2_O_4_:3% Eu^3+^	30.4666	0.2195	**37.10**
SrY_2_O_4_:4% Eu^3+^	30.4966	0.2225	**36.58**
SrY_2_O_4_:5% Eu^3+^	30.5012	0.2337	**34.84**
SrY_2_O_4_:6% Eu^3+^	30.4569	0.2528	**32.20**

**Fig. 3:**
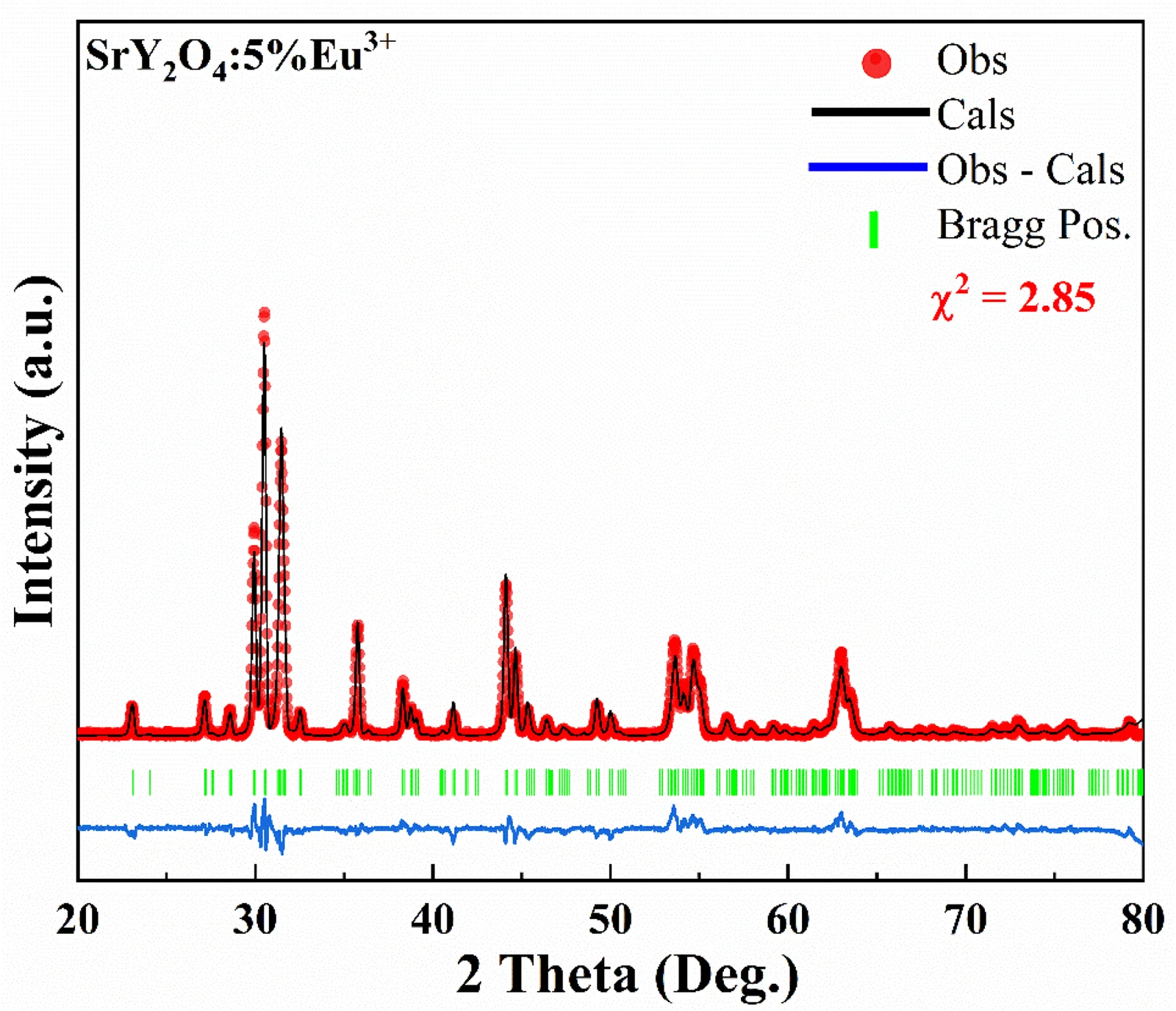
Rietveld refinements of SrY_2_O_4_:5% Eu^3+^ annealed at 1200 °C for 9 h in air.

To systematically evaluate the impact of Eu^3+^ doping concentration on the structural evolution of the host matrix, Rietveld refinement was performed on the SrY_2_O_4_:*x*% Eu^3+^ (*x* = 1.0–6.0) phosphors annealed at 1200 °C. As a representative example, the refinement profile of the 5% Eu^3+^-doped sample is illustrated in Fig. 3 (the remaining patterns are shown in Fig. S1). The extracted lattice parameters (*a*, *b*, *c*), unit cell volume (*V*), and reliability factors (*R*_p_, *R*_exp_, *χ*^2^) are consolidated in Table 2. The low *χ*^2^ values obtained across all samples signify an excellent convergence between the experimental XRD patterns and the calculated models.

**Table 2:** The lattice parameters (*a*, *b*, *c*), unit cell volumes (*V*), and reliability factors (*R*_p_, *R*_exp_, *χ*^2^)

% Eu	*a*/Å	*b* Å	*c*/Å	*V* Å^−3^	*R*_p_/%	*R*_exp_/%	*χ* ^2^
1	10.066	11.902	3.406	408.048	14.50	15.81	**3.80**
2	10.067	11.905	3.407	408.335	15.50	7.05	**2.55**
3	10.070	11.907	3.407	408.407	12.01	15.10	**2.45**
4	10.072	11.911	3.408	408.809	15.10	15.58	**4.07**
5	10.087	11.932	3.414	410.870	17.60	9.13	**2.85**
6	10.075	11.914	3.410	409.272	14.30	13.65	**3.76**

Overall, the lattice constants and unit cell volumes exhibit relatively small variations with increasing Eu^3+^ content, confirming the successful incorporation of Eu^3+^ ions into the SrY_2_O_4_ host lattice. A closer inspection of the refinement data reveals two distinct regimes in the structural behavior. As the Eu^3+^ concentration rises from 1% to 5%, the unit cell volume undergoes a slight expansion. This expansion is attributed to the preferential substitution of the smaller Y^3+^ ions (*r* = 0.90 Å for CN = 6) by the slightly larger Eu^3+^ ions (*r* = 0.947 Å for CN = 6). Conversely, when the doping level reaches 6%, a subtle contraction in the unit cell volume is observed. This inversion strongly suggests that a fraction of Eu^3+^ ions begins to occupy the larger Sr^2+^ sites (*r* = 1.26 Å for CN = 8), thereby inducing a localized lattice contraction. These structural dynamics are in good agreement with previously reported literature on related rare-earth-doped alkaline-earth yttriate systems.^14^

**Fig. 4:**
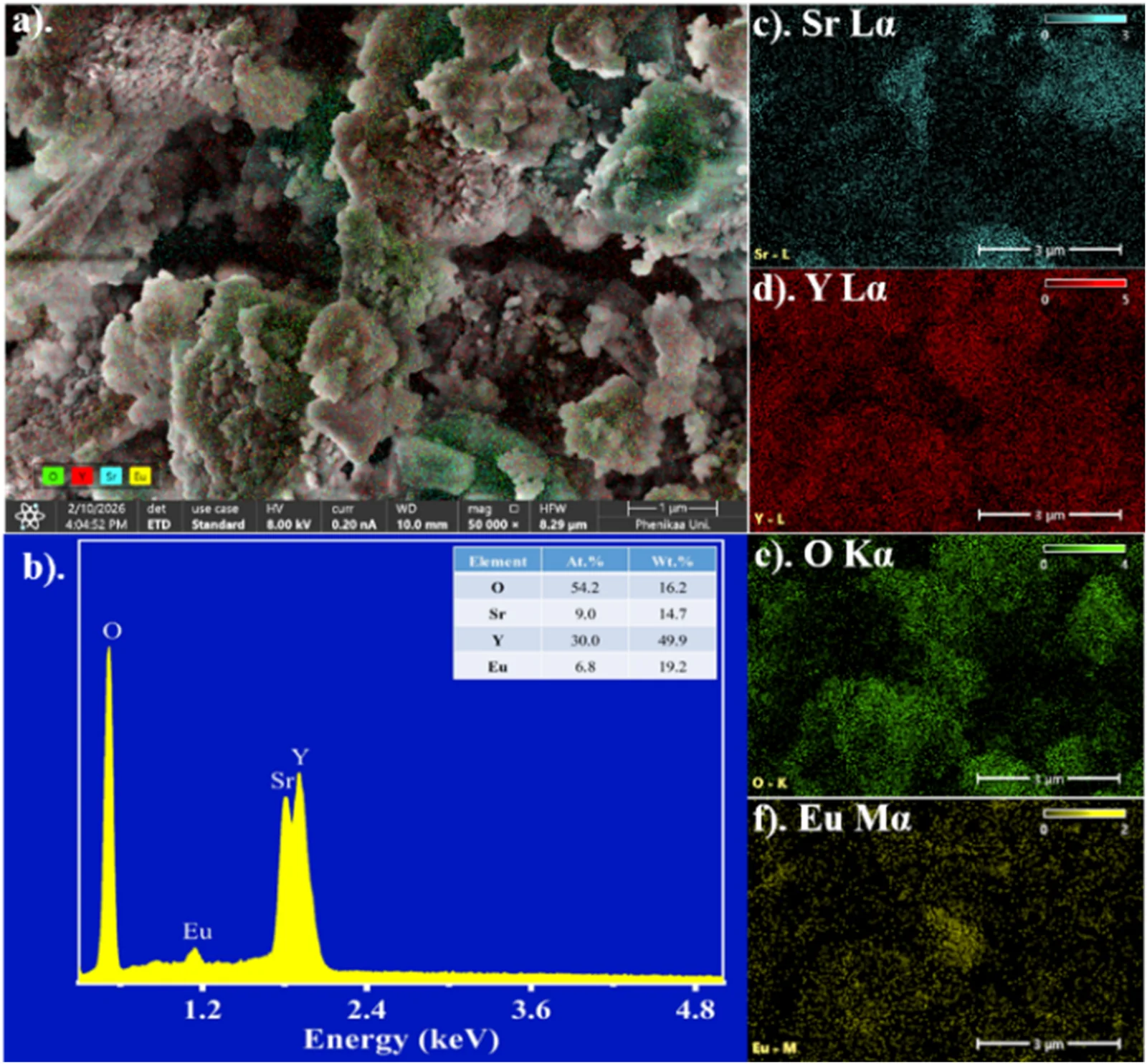
SEM and EDX images of the SrY_2_O_4_:5% Eu^3+^ sample calcined at 1200 °C.

The morphology and elemental composition of the optimized SrY_2_O_4_:5% Eu^3+^ phosphor were further examined by SEM and EDX analyses, as shown in Fig. 4. The SEM image reveals irregularly shaped and agglomerated particles, which are characteristic of phosphors synthesized *via* the conventional solid-state reaction method. The EDX spectrum confirms the presence of Sr, Y, O, and Eu elements without detectable impurity species, demonstrating the successful incorporation of Eu into the SrY_2_O_4_ host lattice. The observed Eu signal further supports the effectiveness of the doping process. Although the measured surface Eu/(Y + Eu) ratio is higher than the nominal composition, EDX analysis probes only a local region of the sample surface. Therefore, additional elemental mapping or high-resolution compositional analyses would be required to conclusively determine the spatial distribution of Eu^3+^ ions within the phosphor particles.

#### 3.1.2. XPS spectra investigation

**Fig. 5:**
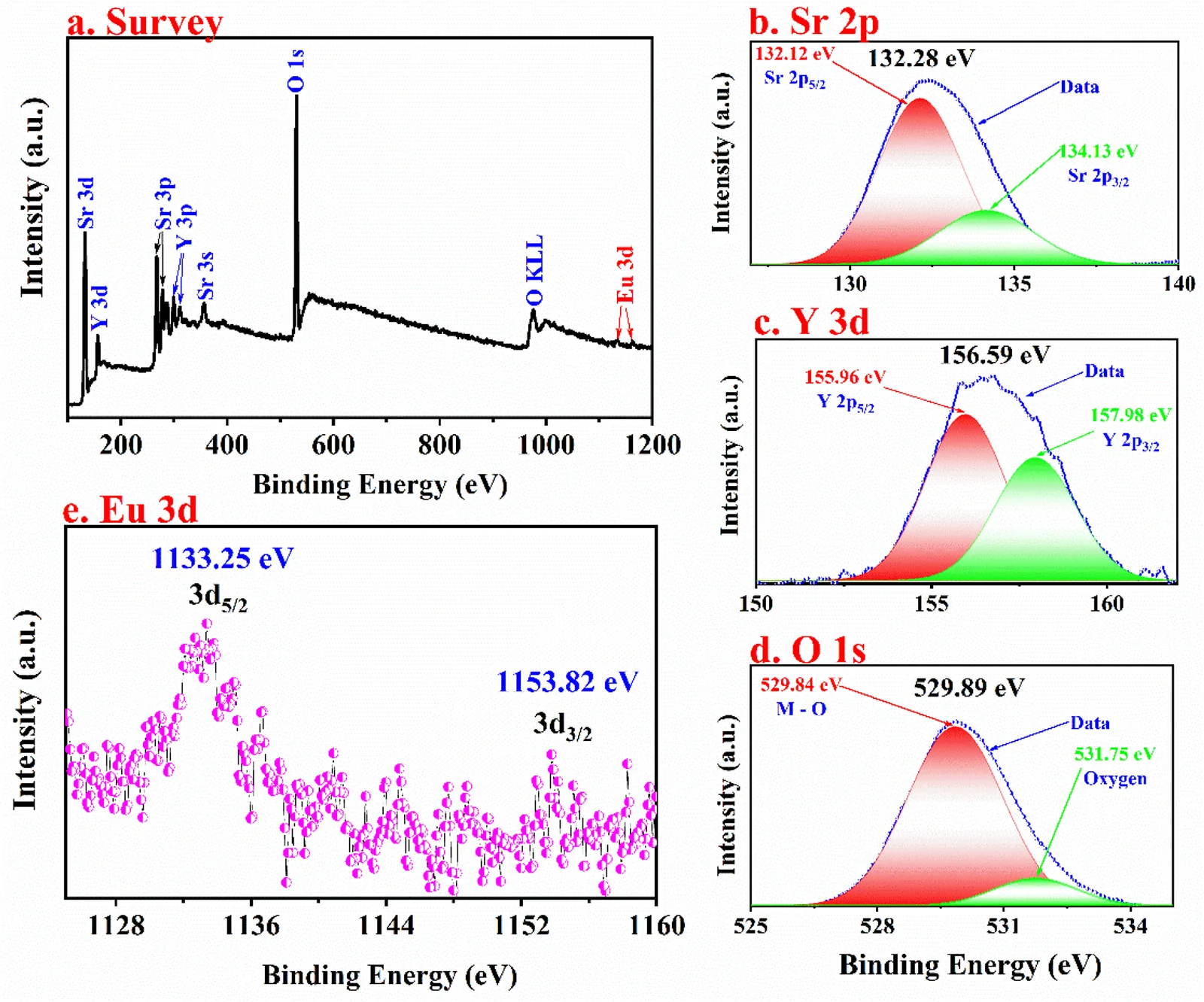
XPS survey spectrum of the 5% Eu-doped SrY_2_O_4_ sample (a), and corresponding high-resolution XPS spectra of the constituent core levels: Sr 2p (b), Y 3d (c), O 1s (d), and Eu 3d (e).

X-ray photoelectron spectroscopy was employed to investigate the elemental composition and chemical states of the SrY_2_O_4_:5% Eu^3+^ phosphor calcined at 1200 °C. The survey spectrum shown in Fig. 5a reveals the presence of Sr, Y, O, and Eu elements without detectable impurity signals, confirming the formation of the Eu-doped SrY_2_O_4_ phosphor.^25^ The high-resolution Sr 2p spectrum (Fig. 5b) exhibits a characteristic spin–orbit doublet centered at approximately 132.28 eV. Deconvolution of the spectrum yields two components located at 132.12 and 134.13 eV, which are assigned to the Sr 3d_5/2_ and Sr 3d_3/2_ states.^25^ The observed binding energies are consistent with those reported for Sr^2+^ ions in oxide lattices, confirming the expected chemical environment of strontium in the SrY_2_O_4_ host.^14^

Fig. 5c presents the high-resolution Y 3d spectrum. Two fitted peaks located at 155.96 and 157.98 eV are attributed to the Y 3d_5/2_ and Y 3d_3/2_ spin–orbit components, respectively. These binding energies are characteristic of Y^3+^ ions and agree well with previously reported values for yttrium-containing oxide materials.^25^ The O 1s spectrum shown in Fig. 5d can be resolved into two components centered at 529.89 and 531.75 eV. The dominant low-binding-energy peak at 529.89 eV is assigned to lattice oxygen (O^2−^) coordinated with metal cations in the Sr–O, Y–O, and Eu–O bonds of the host lattice. The higher-binding-energy component at 531.75 eV is commonly associated with surface oxygen species, hydroxyl groups, oxygen-deficient regions, or adsorbed oxygen on the phosphor surface.^26^ The presence of these two contributions indicates the coexistence of lattice oxygen and surface-related oxygen environments in the synthesized material.

The high-resolution Eu 3d spectrum (Fig. 5e) displays two characteristic peaks located at 1133.25 and 1153.82 eV, corresponding to the Eu 3d_5/2_ and Eu 3d_3/2_ states, respectively.^27^ The measured binding energies are consistent with those reported for Eu^3+^ ions in oxide hosts, confirming that europium is predominantly present in the trivalent oxidation state. The observation of the Eu 3d spin–orbit doublet, together with the absence of impurity phases in the XRD analysis, provides strong evidence for the successful incorporation of Eu^3+^ ions into the SrY_2_O_4_ lattice.

#### 3.1.3. Raman scattering effect

**Fig. 6:**
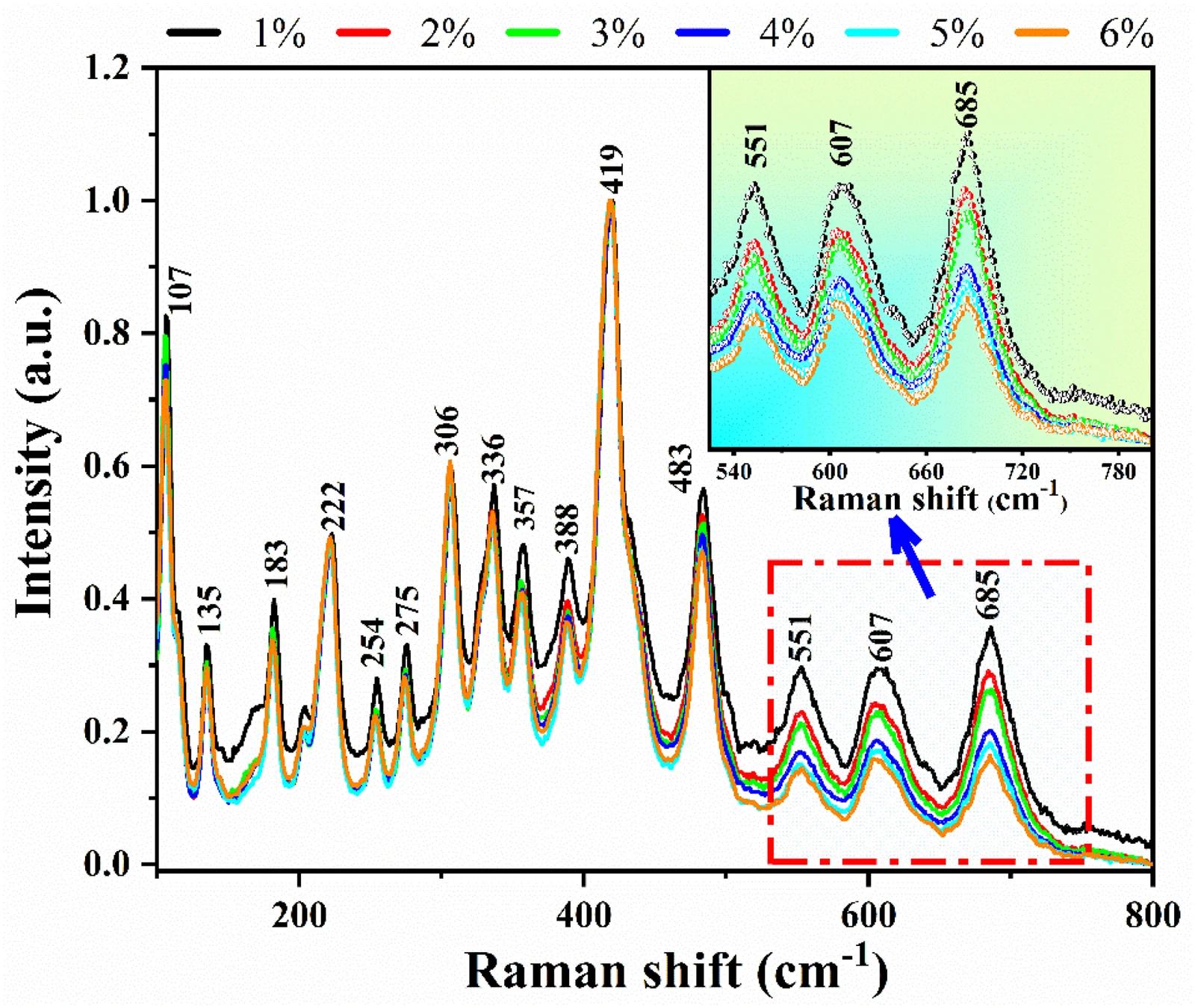
Raman scattering spectra of Eu-doped SrY_2_O_4_ samples excited by a 785 nm laser at room temperature.

Raman spectroscopy was employed to investigate the influence of Eu^3+^ incorporation on the local structure and lattice dynamics of the SrY_2_O_4_ host. Fig. 6 presents the room-temperature Raman spectra of SrY_2_O_4_:*x*% Eu^3+^ (*x* = 1–6 mol%) phosphors recorded under 785 nm laser excitation. As shown in Fig. 6, all samples exhibit nearly identical Raman peak positions over the investigated compositional range, indicating that Eu^3+^ incorporation does not induce any detectable phase transformation or symmetry change in the SrY_2_O_4_ host lattice.^25^ This observation is in good agreement with the XRD results, which confirmed the retention of the orthorhombic *Pnam* structure after doping. The absence of additional Raman bands further suggests that no secondary phases are formed within the detection limit of the measurement. The Raman bands located at approximately 107, 135, 183, and 222 cm^−1^ are attributed to external lattice modes associated with translational vibrations of Sr^2+^ and Y^3+^ cations.^28^ The intermediate-frequency modes centered at 254, 275, 306, 336, 357, 388, and 419 cm^−1^ originate from bending and deformation vibrations of the [YO_6_] polyhedra.^25^ The higher-frequency modes observed at 483, 551, 607, and 685 cm^−1^ are assigned to Y–O stretching vibrations within the SrY_2_O_4_ framework.^29^ Fig. S2 depicts the normalized intensity of the 483 cm^−1^ vibrational mode across this range.

To better evaluate the effect of Eu^3+^ concentration on the local lattice environment, the spectra were normalized with respect to the strongest Raman mode at 419 cm^−1^. As highlighted in the enlarged view of Fig. 6, the low-frequency translational modes exhibit only minor variations with increasing Eu^3+^ concentration. In contrast, the high-frequency Y–O stretching modes located at approximately 551, 607, and 685 cm^−1^ show a gradual decrease in intensity accompanied by noticeable peak broadening.^14^ Such behavior is indicative of increasing local structural disorder and lattice distortion arising from the substitution of Y^3+^ ions (0.90 Å) by the slightly larger Eu^3+^ ions (0.947 Å). The incorporation of Eu^3+^ modifies the local coordination environment around the [YO_6_] polyhedra and introduces fluctuations in Y–O bond lengths and bond angles, resulting in enhanced phonon scattering and broader Raman features.

The observed broadening of the Y–O stretching modes therefore provides additional evidence for the successful incorporation of Eu^3+^ into the SrY_2_O_4_ lattice and the progressive increase in local lattice distortion with increasing dopant concentration. These results are consistent with the XRD analysis, which revealed a slight lattice expansion associated with Eu^3+^ substitution at Y^3+^ sites. Consequently, Raman spectroscopy confirms that Eu^3+^ doping primarily affects the local crystal field environment while preserving the overall orthorhombic framework of the SrY_2_O_4_ host.

#### 3.1.4. Effect of Eu^3+^ ion concentration on infrared absorption

**Fig. 7:**
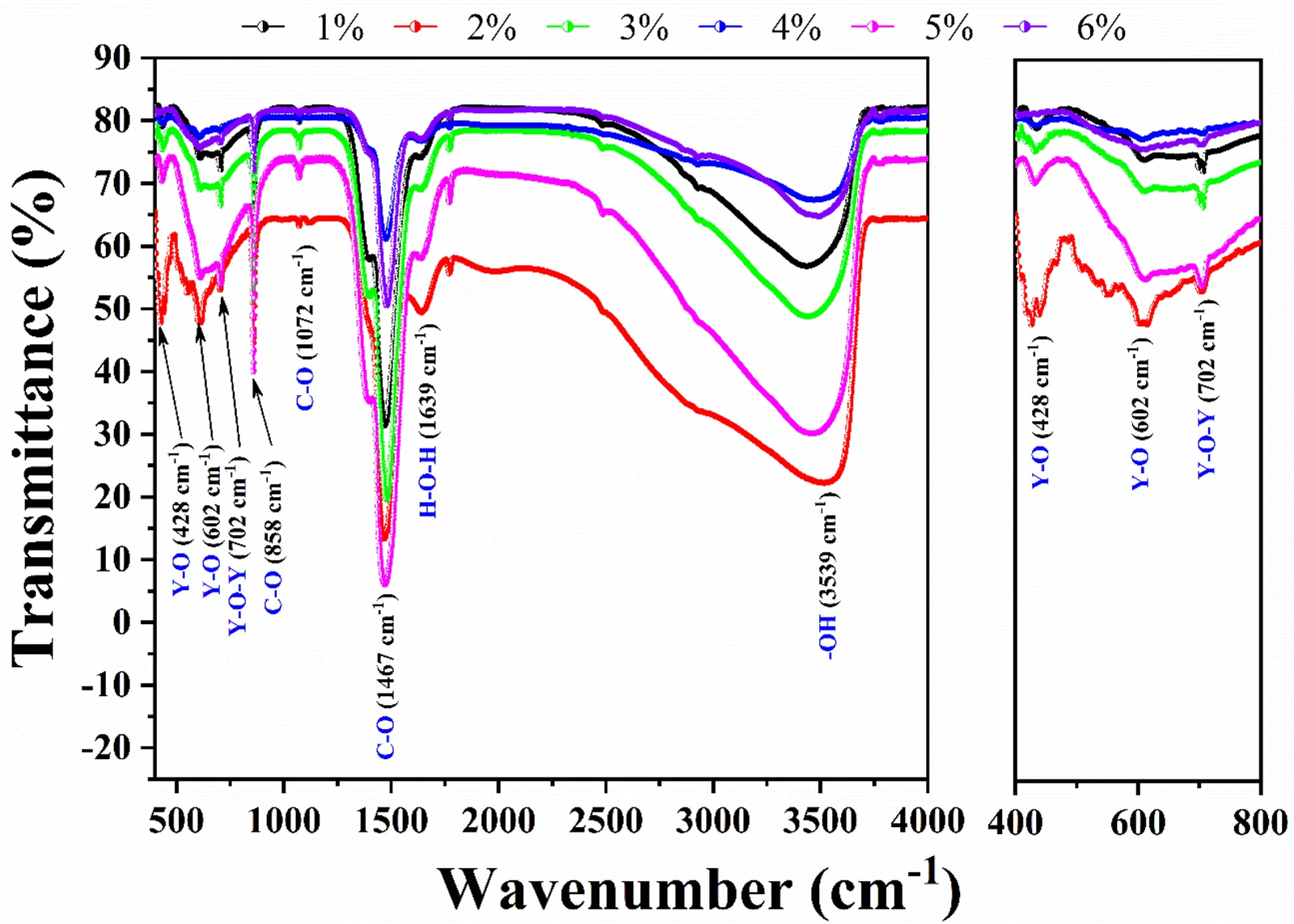
FT-IR of SrY_2_O_4_:*x*Eu^3+^ samples (*x* = 1–6 mol%) calcined at 1200 °C measured at room temperature.

Fourier-transform infrared spectroscopy was employed to investigate the effect of Eu^3+^ concentration on the bonding environment and local structure of the SrY_2_O_4_ host lattice. Fig. 7 presents the room-temperature FTIR spectra of SrY_2_O_4_:*x*% Eu^3+^ (*x* = 1–6 mol%) phosphors calcined at 1200 °C. All samples exhibit similar absorption features, indicating that the incorporation of Eu^3+^ does not significantly alter the fundamental crystal structure of the SrY_2_O_4_ host. The spectra display characteristic absorption bands centered at approximately 428, 602, 702, 858, 1027, 1476, 1639, and 3539 cm^−1^. The low-wavenumber bands at 428 and 602 cm^−1^ are assigned to Y–O vibrational modes within the [YO_6_] polyhedra, whereas the absorption band at approximately 702 cm^−1^ is attributed to Y–O–Y bridging vibrations characteristic of the SrY_2_O_4_ framework.^15,25,30^ The persistence of these bands for all compositions confirms the structural stability of the orthorhombic host lattice upon Eu^3+^ doping.

The absorption bands observed at approximately 858, 1027, and 1476 cm^−1^ are associated with carbonate species and atmospheric CO_2_ adsorbed on the sample surface, while the bands at 1639 and 3539 cm^−1^ originate from the bending and stretching vibrations of adsorbed water molecules and surface hydroxyl groups.^10^ Such features are commonly observed in oxide phosphors exposed to ambient conditions. Although no new absorption bands appear after Eu^3+^ incorporation, noticeable changes in the intensity and line shape of the Y–O-related bands, particularly those centered at 602 and 702 cm^−1^, can be observed with increasing Eu^3+^ concentration. These variations suggest that Eu^3+^ ions influence the local bonding environment of the [YO_6_] network. Because Eu^3+^ (0.947 Å) possesses a slightly larger ionic radius than Y^3+^ (0.90 Å), its substitution into Y^3+^ sites can introduce local lattice distortion and modify the Y–O bond lengths and bond angles. Consequently, subtle changes in the vibrational characteristics of the lattice are observed in the FTIR spectra.

The FTIR results therefore support the conclusions drawn from the XRD and Raman analyses, namely that Eu^3+^ ions are successfully incorporated into the SrY_2_O_4_ lattice without altering its orthorhombic crystal structure, while inducing local structural distortions around the rare-earth sites. Such modifications of the local crystal field environment are expected to influence the optical and luminescence properties of the phosphors.

#### 3.1.5. High-resolution transmission electron microscopy

**Fig. 8:**
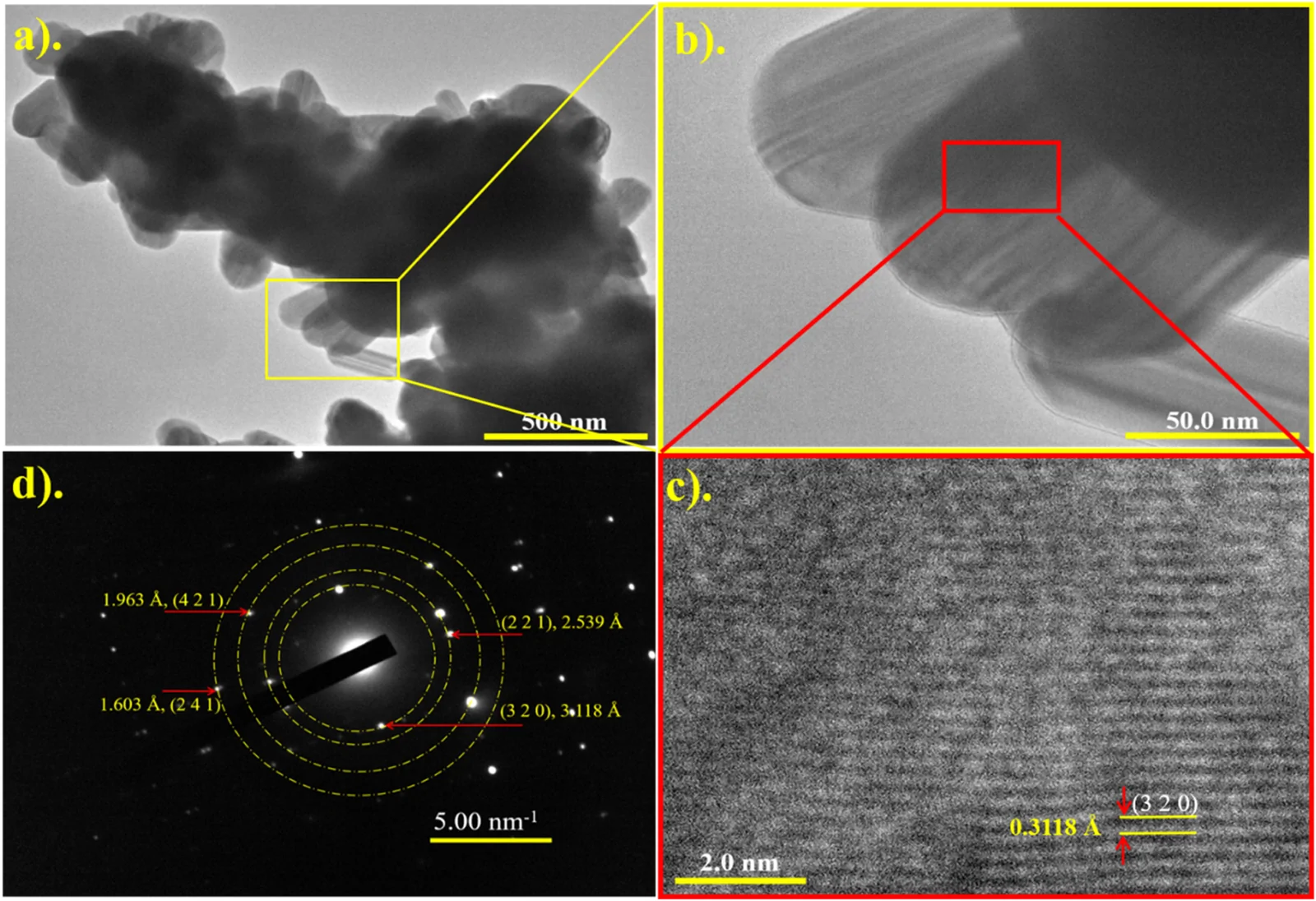
Microstructural analysis of the SrY_2_O_4_:6%Eu^3+^ sample: (a and b) TEM images at different magnifications, (c) HRTEM image showing lattice fringes of the (3 2 0) plane, and (d) SAED pattern with indexed rings.

To further investigate the microstructure and crystallinity of the synthesized phosphors, high-resolution transmission electron microscopy (HRTEM) and selected-area electron diffraction (SAED) analyses were performed on the SrY_2_O_4_:6% Eu^3+^ sample calcined at 1200 °C. Representative TEM, HRTEM, and SAED images are shown in Fig. 8. The low-magnification TEM image (Fig. 8a) reveals that the phosphor particles are composed of agglomerated nanocrystalline grains, which is consistent with the morphology observed in the SEM analysis. The particle aggregation is typical of oxide phosphors prepared by high-temperature solid-state reactions. The corresponding HRTEM image (Fig. 8b and c) exhibits well-resolved lattice fringes, confirming the high crystallinity of the synthesized material. The measured interplanar spacing is approximately 3.118 Å, which can be indexed to the (320) crystallographic plane of orthorhombic SrY_2_O_4_.^10,25,31^ This value agrees well with the XRD results and further confirms the formation of the SrY_2_O_4_ phase. The SAED pattern shown in Fig. 8d consists of a series of concentric diffraction rings, indicating that the sample possesses a polycrystalline nature with randomly oriented crystallites. The measured *d*-spacings of approximately 3.118, 2.539, 1.963, and 1.693 Å can be assigned to the (320), (221), (421), and (431) planes of orthorhombic SrY_2_O_4_, respectively.^10,25,31^

The good agreement between the SAED indexing, HRTEM lattice fringes, and XRD analysis provides strong evidence for the successful formation of highly crystalline SrY_2_O_4_:6% Eu^3+^ sample. The HRTEM and SAED results therefore corroborate the XRD findings, confirming that Eu^3+^ incorporation does not alter the orthorhombic crystal structure of SrY_2_O_4_. Instead, the dopant ions are accommodated within the host lattice while maintaining the long-range structural order of the material.

### 3.2. Optical properties

#### 3.2.1. UV-vis analysis

**Fig. 9:**
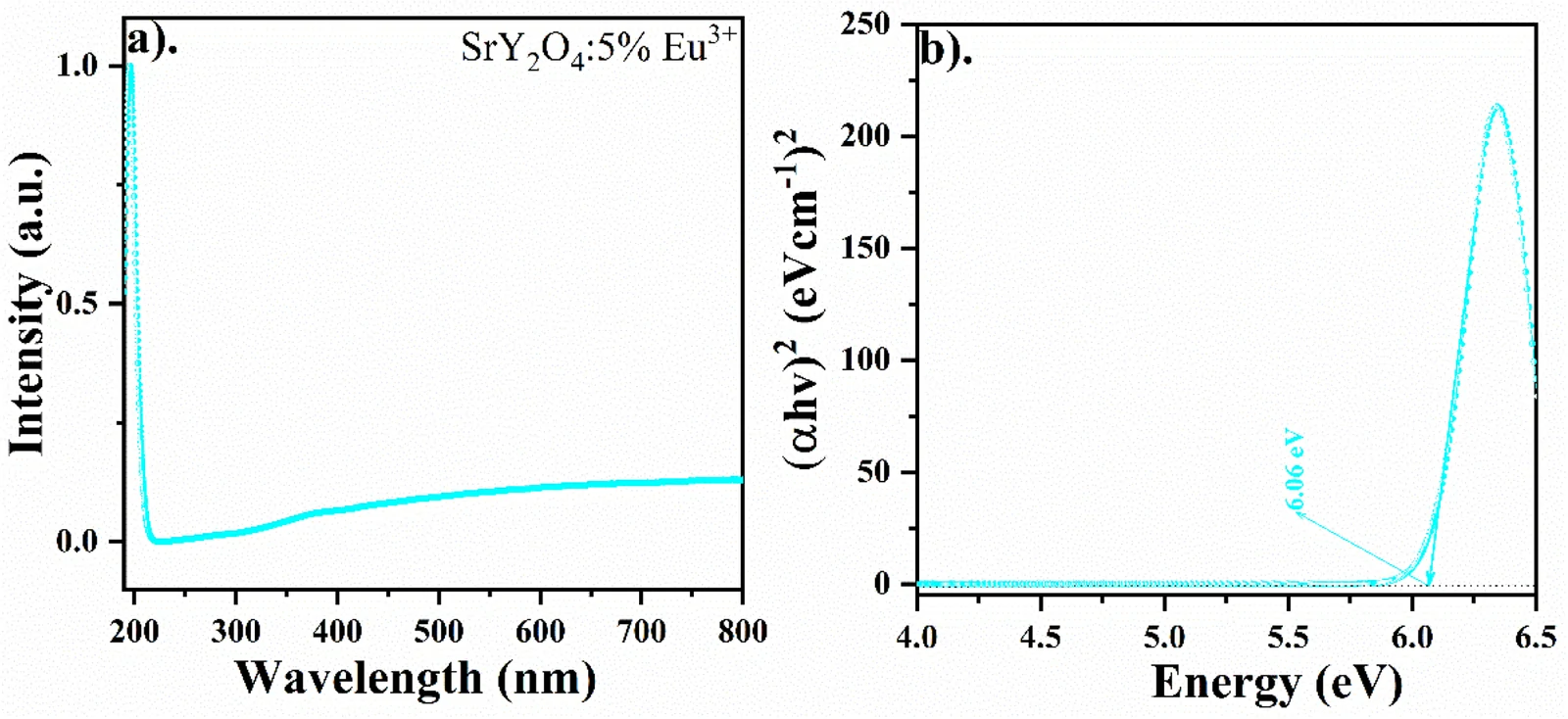
(a) UV-vis absorption spectrum and (b) Tauc plot of the SrY_2_O_4_:6%Eu^3+^ phosphor annealed at 1200 °C for 9 h.

The optical absorption properties of the SrY_2_O_4_:*x*% Eu^3+^ (*x* = 1–6 mol%) phosphors were investigated by UV-vis spectroscopy to evaluate the influence of Eu^3+^ concentration on the electronic structure of the host lattice. Fig. 9a shows the absorption spectra of the synthesized samples. All compositions exhibit strong absorption in the deep-ultraviolet region with an absorption edge located below 250 nm, which is characteristic of the wide-bandgap SrY_2_O_4_ host. The intense absorption band centered at approximately 192 nm can be attributed to host-lattice electronic transitions from the valence band to the conduction band. The absence of strong absorption in the visible region indicates that the optical response is primarily governed by the host lattice rather than by direct Eu^3+^ absorption. A gradual shift of the absorption edge toward longer wavelengths is observed with increasing Eu^3+^ concentration, suggesting a modification of the electronic structure induced by dopant incorporation. To quantify this effect, the optical band-gap energy (*E*_g_) was estimated using the Tauc relation:^32^

3 *αhυ* = *A*(*hυ* − *E*_g_)^*n*^

where *α* is the absorption coefficient, *hν* is the incident photon energy, *A* is a proportionality constant, and *n* depends on the nature of the optical transition. For the SrY_2_O_4_, host matrix, a direct allowed transition was adopted, which corresponds to *n* = ½ in the Tauc relation. Consequently, the optical band gap (*E*_g_) was determined by extrapolating the linear intercept of the (*αhν*)^2^*versus hv* plot onto the photon energy axis, as shown in Fig. 9. The calculated band-gap energies for SrY_2_O_4_:5% Eu^3+^ (the remaining patterns are shown in Fig. S3) are 6.13, 6.11, 6.09, 6.08, 6.06, and 5.93 eV, respectively. A systematic decrease in *E*_g_ is therefore observed with increasing Eu^3+^ concentration. This reduction can be attributed to the introduction of localized electronic states associated with Eu^3+^ ions and the increasing lattice distortion resulting from the substitution of Y^3+^ (0.90 Å) by the slightly larger Eu^3+^ ions (0.947 Å). As dopant concentration increases, the perturbation of the crystal potential becomes more pronounced, leading to the formation of defect-related states and band-tail broadening near the band edges. Consequently, the effective optical band gap decreases and the absorption edge shifts toward lower photon energies.

The observed band-gap narrowing is consistent with the structural analyses obtained from XRD and Raman spectroscopy, which revealed a slight lattice expansion and increasing local disorder with increasing Eu^3+^ content.

The gradual modification of the electronic structure is expected to influence the excitation and energy-transfer processes of Eu^3+^ ions and therefore plays an important role in determining the luminescence performance of the SrY_2_O_4_:Eu^3+^ phosphors.

#### 3.2.2. Luminescence of materials

**Fig. 10:**
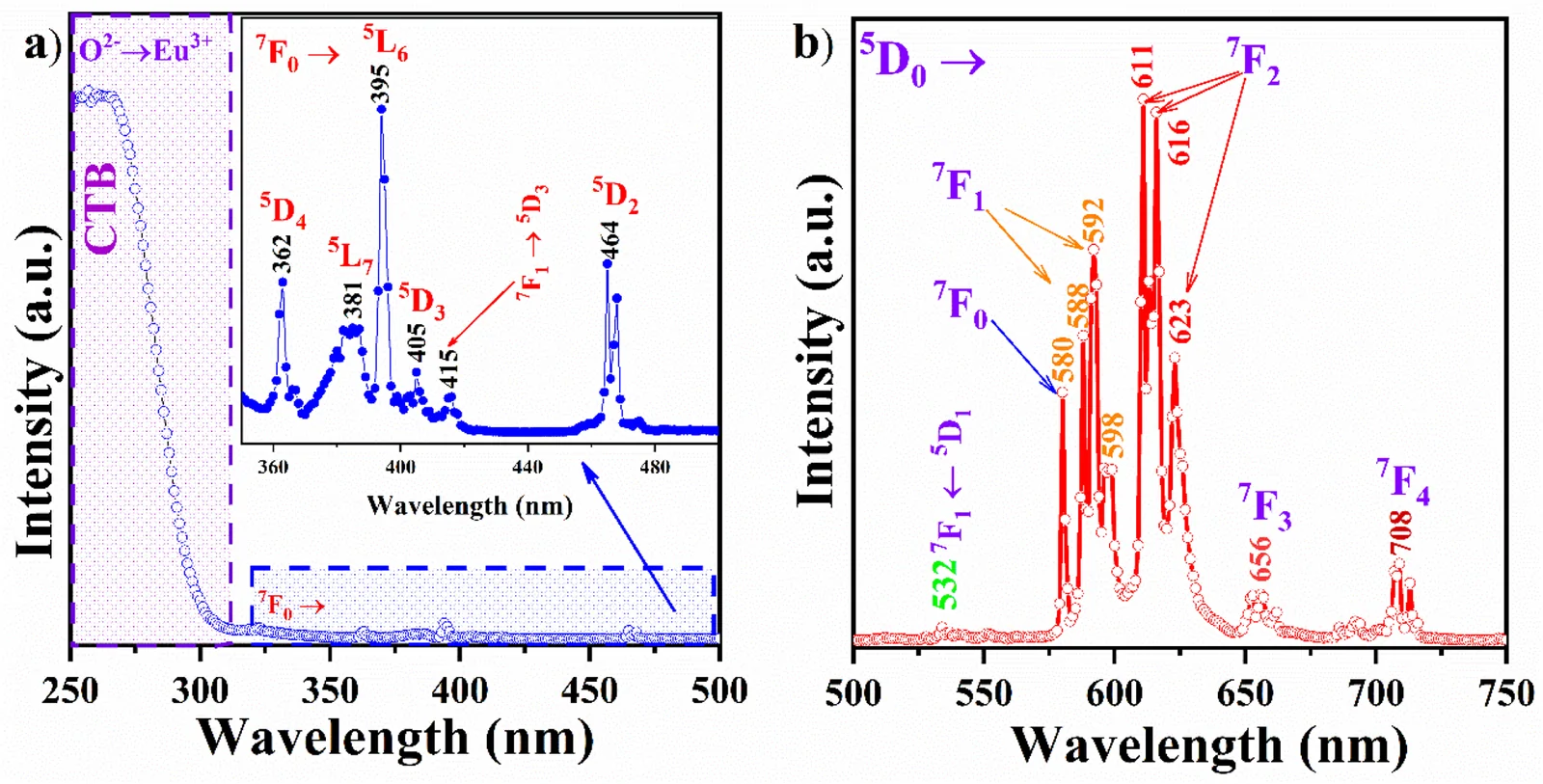
(a) PLE, and (b) PL of SrY_2_O_4_:5% Eu sample heated to 1200 °C measured at room temperature.

The photoluminescence excitation and emission spectra of the SrY_2_O_4_:5% Eu^3+^ phosphor measured at room temperature are presented in Fig. 10. The PLE spectrum monitored at 611 nm (Fig. 10a) exhibits a broad and intense excitation band in the ultraviolet region with a maximum centered at approximately 254 nm. This broad band is attributed to the charge-transfer band (CTB) associated with the electronic transition from the 2p orbital of O^2−^ ligands to the 4f shell of Eu^3+^ ions within the SrY_2_O_4_ host lattice.^23,33^ This excitation process follows a transient redox mechanism, wherein the absorption of a high-energy photon temporarily reduces Eu^3+^ to Eu^2+^ while oxidizing the ligand to O^−^. The subsequent non-radiative relaxation and electron–hole recombination efficiently populate the upper excited ^5^D_J_ energy levels of the Eu^3+^ activators, leading to the characteristic emission. In addition to the CTB, several sharp excitation peaks are observed in the near-ultraviolet and visible regions. These bands originate from the characteristic intra-4f transitions of Eu^3+^ ions from the ^7^F_0_ ground state to higher excited states. The excitation peaks located at approximately 362, 395, and 464 nm are assigned to the ^7^F_0_ → ^5^D_4_, ^7^F_0_ → ^5^L_6_, and ^7^F_0_ → ^5^D_2_ transitions, respectively.^23,33^ Among these transitions, the ^7^F_0_ → ^5^L_6_ transition at 395 nm exhibits the highest intensity, indicating that the phosphor can be effectively matched with and excited by near-ultraviolet LED chips within the practical context of lanthanide-activated solid-state lighting material. The corresponding PL spectrum recorded under 395 nm excitation is shown in Fig. 10b. The emission spectrum consists of a series of narrow bands characteristic of Eu^3+^ ions, arising from radiative transitions from the ^5^D_0_ excited state to the ^7^F_J_ (*J* = 0–4) multiplet levels.^34–36^

The emission peaks centered at approximately 580, 593, 611, 616, 623, 652, and 708 nm are assigned to the ^5^D_0_ → ^7^F_0_, ^5^D_0_ → ^7^F_1_, ^5^D_0_ → ^7^F_2_, ^5^D_0_ → ^7^F_2_, ^5^D_0_ → ^7^F_2_, ^5^D_0_ → ^7^F_3_, and ^5^D_0_ → ^7^F_4_ transitions, respectively. The detection of a distinct ^5^D_0_ → ^7^F_0_ emission peak at 580 nm strongly indicates that Eu^3+^ dopants reside at the 4c Wyckoff positions of the orthorhombic SrY_2_O_4_ lattice (space group *Pnam*). Both the Y^3+^ sites (coordinated in YO_6_ octahedra) and the Sr^2+^ sites share this 4c position, which features a local point symmetry of *C*_s_. The non-centrosymmetric nature of this *C*_s_ site symmetry accounts for the lifting of the quantum selection rules, thereby inducing the observed 0–0 transition. Among these emissions, the ^5^D_0_ → ^7^F_2_ transition in the red spectral region is dominant, producing intense red luminescence. The significantly higher intensity of the hypersensitive, forced-electric dipole ^5^D_0_ → ^7^F_2_ transition compared with the magnetic-dipole allowed ^5^D_0_ → ^7^F_1_ transition indicates that the Eu^3+^ ions occupy non-centrosymmetric lattice sites (*C*_s_ point symmetry) in the orthorhombic SrY_2_O_4_ lattice, which induces a strong crystal-field perturbation that relaxes the parity selection rule. The dominance of this hypersensitive transition is also responsible for the high color purity of the emitted red light, which is highly desirable for phosphor-converted white LED applications.

**Fig. 11:**
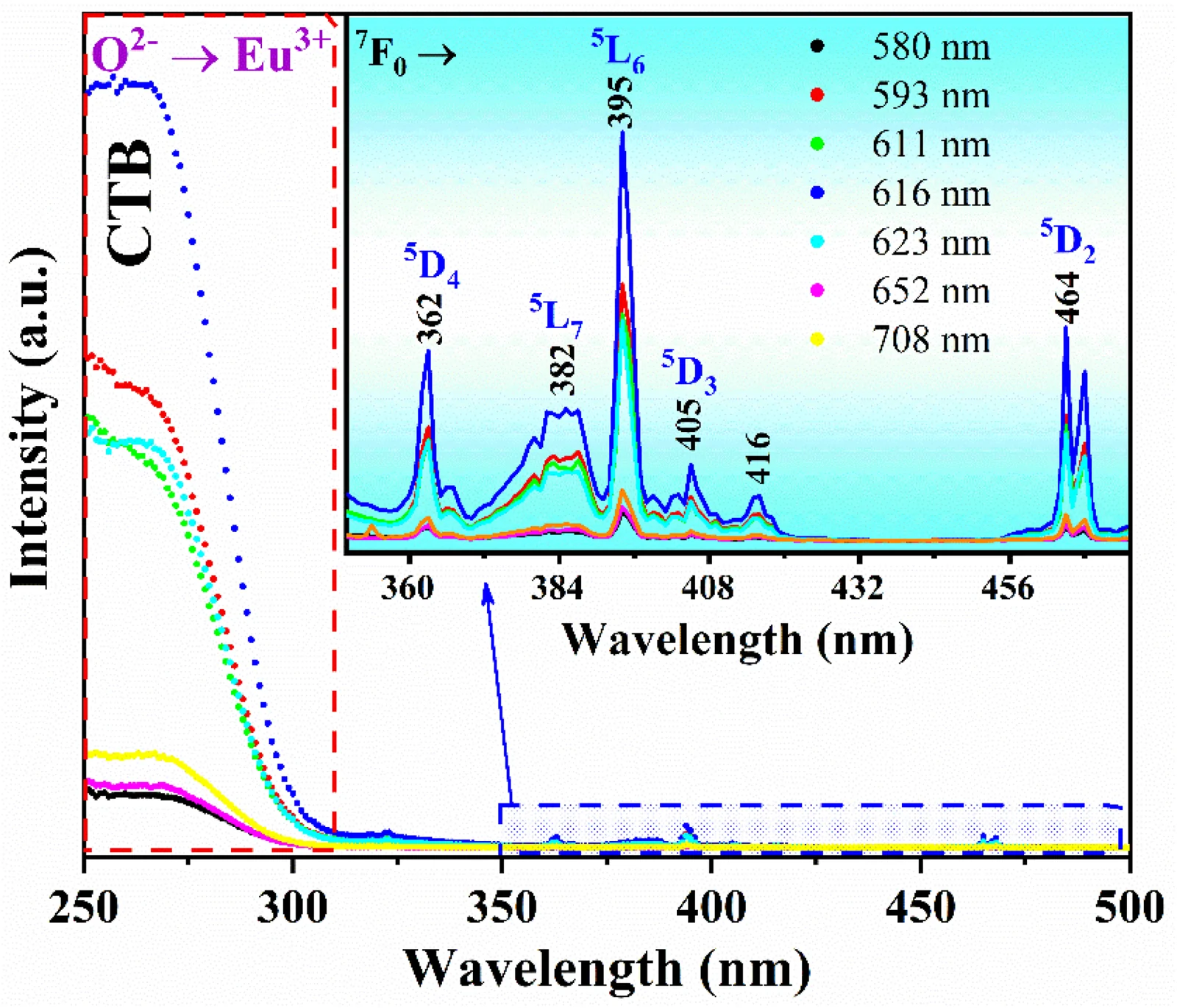
PLE of SrY_2_O_4_:5% Eu sample heated to 1200 °C corresponding to different emission wavelengths.

To further investigate the excitation behavior associated with different emission transitions, PLE spectra were recorded by monitoring emission wavelengths at 580, 593, 611, 616, 623, 652, and 708 nm, as shown in Fig. 11. All monitored emissions exhibit similar excitation profiles consisting of the broad O^2−^ → Eu^3+^ CTB and the characteristic Eu^3+^ intra-4f excitation bands. These nearly identical excitation peak positions indicate that these emission transitions indeed originate from the same Eu^3+^ luminescent centers. However, the excitation intensity varies with the monitored emission wavelength. The strongest excitation response is observed for the emission monitored at 611 nm, corresponding to the dominant, hypersensitive forced-electric dipole ^5^D_0_ → ^7^F_2_ transition.

This result confirms that the energy absorbed through both the CTB and the intra 4f excitation channels is effectively channeled to the ^5^D_0_ emitting level prior to radiative relaxation. The prominent near-ultraviolet excitation, the highly effective charge-transfer sensitization process, and the intense red emission dominated by the forced-electric dipole ^5^D_0_ → ^7^F_2_ transition demonstrate the high potential of SrY_2_O_4_:Eu^3+^ phosphors as red-emitting components for warm-white light-emitting diode applications.

#### 3.2.3. Excitation-wavelength-dependent emission and concentration quenching behavior

**Fig. 12:**
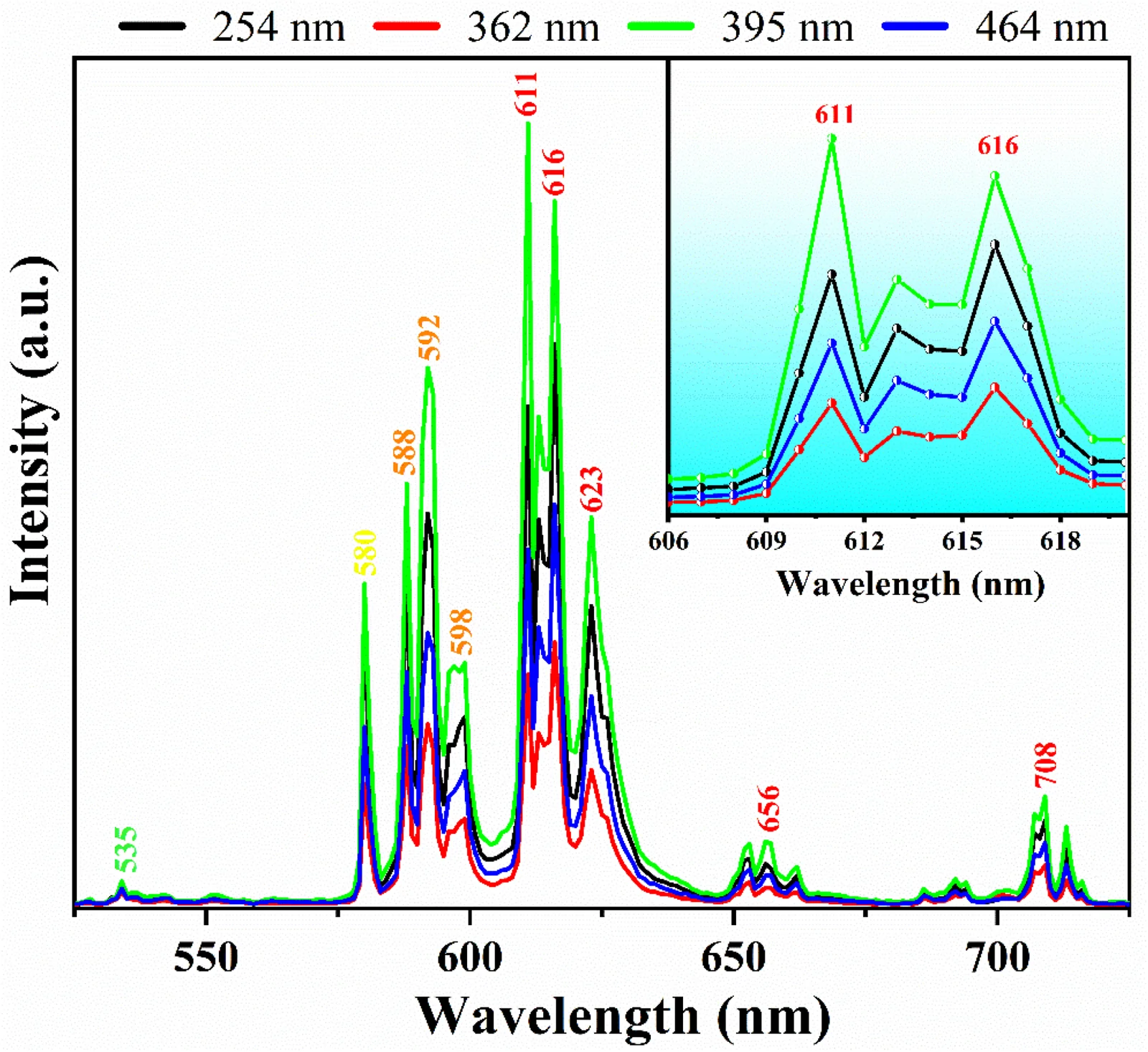
Luminescence spectrum of sample SrY_2_O_4_:5% Eu^3+^ heated to 1200 °C corresponding to different excitation wavelengths.

Based on the PLE results, the photoluminescence properties of the SrY_2_O_4_:5% Eu^3+^ phosphor were further investigated under excitation wavelengths of 254, 362, 395, and 464 nm. The corresponding emission spectra are shown in Fig. 12. Regardless of the excitation wavelength, all spectra exhibit the characteristic emission bands of Eu^3+^ ions arising from the ^5^D_0_ → ^7^F_J_ (*J* = 0–4) transitions. The emission peak positions remain essentially unchanged across different excitation pathways. This stability indicates that the local crystal-field strength and coordination environment around the activator ions are highly stable. Because the even-parity components of the crystal-field Hamiltonian which govern the energy level positions and Stark splitting remain unaffected by the excitation mode, the Eu^3+^ ions occupy sites with identical local symmetry irrespective of how they are populated.

The strongest emission bands are observed in the orange-red spectral region, particularly at approximately 593 nm (^5^D_0_ → ^7^F_1_) and 611–623 nm (^5^D_0_ → ^7^F_2_). Among the investigated excitation wavelengths, excitation at 395 nm (^7^F_0_ → ^5^L_6_) produces the highest emission intensity, confirming the excellent spectral matching of the phosphor with near-ultraviolet LED chips. In addition, intense luminescence is also obtained under 464 nm excitation 464 nm excitation, corresponding to the ^7^F_0_ → ^5^D_2_ transition, demonstrating the potential applicability of the material for blue-LED-pumped phosphor-converted white LEDs. The persistence of strong red emission under both near-ultraviolet and blue excitation highlights the versatility of the SrY_2_O_4_:5% Eu^3+^ phosphor for solid-state lighting applications within the context of trivalent lanthanide-activated luminescent materials.

**Fig. 13:**
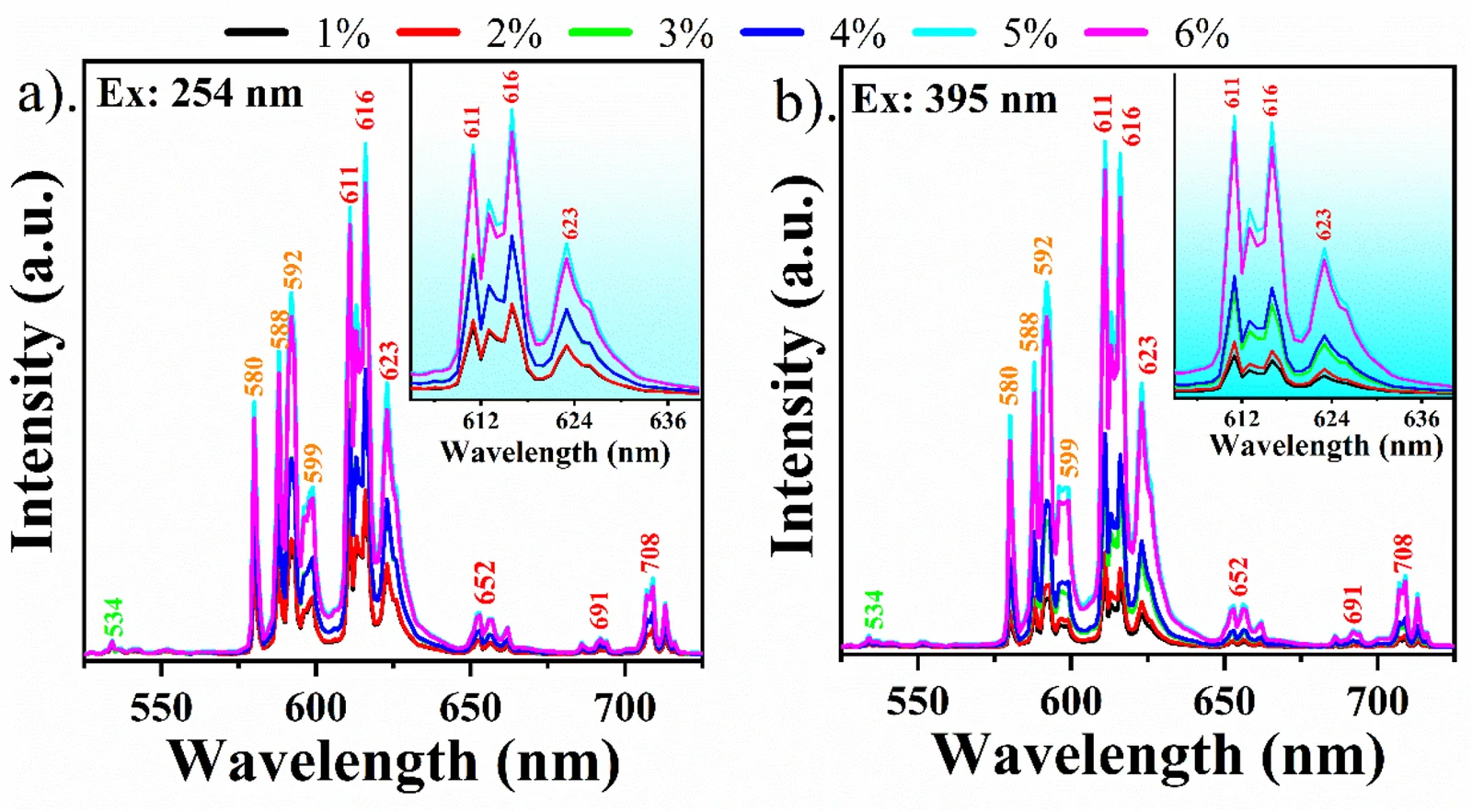
PL of sample SrY_2_O_4_:*x*% Eu heated to 1200 °C corresponding to excitation wavelengths of 254 (a), and 395 nm (b).

To investigate the influence of Eu^3+^ concentration on the luminescence behavior, emission spectra of SrY_2_O_4_:*x*% Eu^3+^ (*x* = 1–6 mol%) phosphors were recorded under 254 and 395 nm excitation, as shown in Fig. 13. For both excitation wavelengths, the spectral profiles remain unchanged with increasing Eu^3+^ concentration, indicating that the emission originates from the same radiative transitions of Eu^3+^ ions. However, the emission intensity strongly depends on the activator concentration. As the Eu^3+^ concentration increases from 1 to 5 mol%, the emission intensity gradually increases due to the increasing number of luminescent centers incorporated into the SrY_2_O_4_ host lattice. The maximum emission intensity is achieved at an Eu^3+^ concentration of 5 mol%, indicating the optimum activator content for this phosphor system. Further increasing the Eu^3+^ concentration to 6 mol% results in a noticeable decrease in emission intensity, revealing the onset of concentration quenching.

The concentration quenching phenomenon is generally associated with enhanced non-radiative energy transfer among neighboring Eu^3+^ ions when the average Eu–Eu distance becomes sufficiently small. At high activator concentrations, excitation energy can migrate between Eu^3+^ centers and be dissipated through non-radiative pathways, thereby reducing the radiative emission efficiency. The absence of significant spectral broadening or changes in emission profiles suggests that the quenching mechanism primarily originates from Eu^3+^–Eu^3+^ energy migration rather than the formation of new luminescent centers. Consequently, the optimum Eu^3+^ concentration for achieving maximum red emission in the SrY_2_O_4_ host is determined to be 5 mol%. The similar concentration dependence observed under both 254 and 395 nm excitation indicates that the host-to-activator charge-transfer process and the direct Eu^3+^ excitation channels ultimately populate the same ^5^D_0_ emitting level. Therefore, regardless of the excitation route, the luminescence efficiency of the phosphor is governed primarily by the concentration-dependent interaction between Eu^3+^ ions.

#### 3.2.4. Asymmetry ratio and energy-transfer mechanism

The luminescence behavior of Eu^3+^ ions is strongly influenced by the local crystal-field environment surrounding the activator ions. In particular, the forced-electric dipole transition ^5^D_0_ → ^7^F_2_ is highly sensitive to the local symmetry of the Eu^3+^ site, whereas the magnetic-dipole transition ^5^D_0_ → ^7^F_1_ is comparatively insensitive to changes in the crystal field. Therefore, the ratio between the integrated emission intensities of these two transitions, commonly referred to as the asymmetry ratio (*R*), can be used to evaluate the local environment and covalency around Eu^3+^ ions. The asymmetry ratio was calculated according to:4
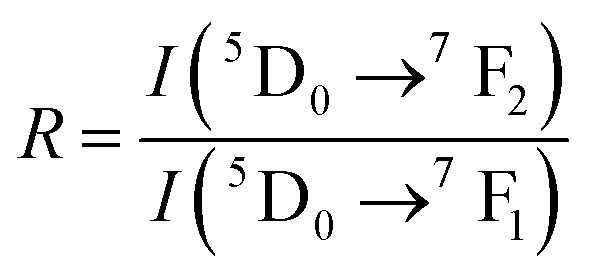
where *I*(^5^D_0_ → ^7^F_2_) and *I*(^5^D_0_ → ^7^F_1_) represent the integrated emission intensities of the red and orange emission bands, respectively.

The calculated asymmetry ratio (*R*) values are summarized in Table 4. As the Eu^3+^ concentration increases from 1 to 4 mol%, *R* increases marginally from 1.770 to 1.886, followed by a slight decrease at higher doping levels. The initial upward trend in *R* suggests a progressive, yet modest, enhancement in the local crystal-field asymmetry and a slight increase in the covalency of the Eu–O bond. This behavior is primarily driven by the substitution of Y^3+^ sites within the YO_6_ octahedra by the slightly larger Eu^3+^ ions, inducing a minor local lattice distortion. Notably, the overall values of *R* remain consistently low (around 1.8), indicating that although the Eu^3+^ ions reside at the non-centrosymmetric 4c Wyckoff sites (with *C*_s_ site symmetry), the host coordination polyhedra are only minimally distorted. The local environment of the luminescent centers thus retains a quasi-centrosymmetric character throughout the investigated concentration range, restraining the forced-electric dipole transition (^5^D_0_ → ^7^F_2_) from heavily dominating the spectrum. These spectral features are in excellent agreement with the XRD, Raman, and FTIR analyses, which collectively confirm the preservation of the orthorhombic SrY_2_O_4_ host framework alongside only minor local structural perturbations. Beyond the dominant emissions originating from the ^5^D_0_ state, a weak emission band centered at approximately 535 nm assigned to the ^5^D_1_ → ^7^F_1_ transition is resolved in the photoluminescence spectra.^37^ The emergence of this ^5^D_1_ emission indicates that a fraction of the excited Eu^3+^ ions undergo direct radiative relaxation to the ground multiplets before undergoing complete, non-radiative multi-phonon relaxation to the lower-lying ^5^D_0_ state. Such transitions from higher excited states are typically sustained in hosts with high crystallinity and low defect/quenching center densities, which restrict non-radiative loss pathways.

To evaluate the influence of Eu^3+^ concentration on the luminescence dynamics and deactivation pathways of the ^5^D_1_ state, the intensity ratio of the ^5^D_1_ → ^7^F_1_ emission at 535 nm to the dominant ^5^D_0_ → ^7^F_2_ emission at 611 nm was calculated and listed in Table 4. For the sample containing 1 mol% Eu^3+^, this ratio is approximately 8.04%, indicating that a measurable fraction of the excited ions undergoes direct radiative relaxation from the ^5^D_1_ level. As the Eu^3+^ concentration increases, this ratio decreases continuously to 3.06% for the 6 mol% Eu^3+^ sample.

While the intrinsic, concentration-independent multiphonon relaxation (MPR) consistently contributes to the deactivation of the ^5^D_1_ level, the continuous decline in the ^5^D_1_/^5^D_0_ ratio with increasing doping levels is primarily driven by inter-ionic cross-relaxation (CR). This concentration-dependent energy transfer (ET) process occurs *via* the resonant pathway:^14,37^^5^D_1_ + ^7^F_0_ → ^5^D_0_ + ^7^F_3_which effectively depopulates the ^5^D_1_ state and feeds the lower-lying ^5^D_0_ emitting level. As the average Eu–Eu distance decreases with higher activator concentration, multipolar interactions become increasingly prominent, accelerating this CR process. Consequently, the relative intensity of the ^5^D_0_ → ^7^F_2_ emission is enhanced at the expense of the ^5^D_1_ emission contribution.

This concentration dependent CR dynamics provides clear evidence of increasing inter-ionic interactions and is closely correlated with the overall concentration-quenching behavior observed at Eu^3+^ concentrations above 5 mol%. Together, the asymmetry-ratio analysis and the evolution of the ^5^D_1_/^5^D_0_ emission intensity ratio demonstrate that Eu^3+^ incorporation modifies both the local crystal-field environment and the energy-transfer dynamics within the SrY_2_O_4_ host lattice, thereby governing the overall luminescent efficiency of the phosphor.

#### 3.2.5. Concentration quenching mechanism

To elucidate the concentration-quenching mechanism in theSrY_2_O_4_:*x*% Eu^3+^ phosphors, the critical transfer distance (*R*_c_) between neighboring Eu^3+^ ions was estimated using Blasse's equation:^11^5
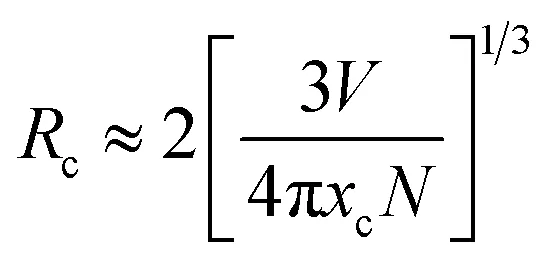
where *x*_c_ is the critical activator concentration, *V* is the unit cell volume, and *N* is the coordination number of available cation sites occupied by the activator ions in a unit cell. For the present SrY_2_O_4_:*x*% Eu^3+^ system, the values (*x*_c_ = 0.05), (*V* = 410.870) Å^3^, and (*N* = 8) (Y^3+^sites per unit cell in space group *Pnma*), the calculated critical distance (*R*_c_) is approximately 12.52 Å.

According to Blasse's criterion, exchange interactions typically dominate only when the interatomic distance between neighboring activators is less than 5 Å. Because the calculated *R*_c_ value 12.52 Å significantly exceeds this threshold, exchange interaction can be ruled out as the primary mechanism for concentration quenching. Instead, the process is governed by electric multipolar interactions between adjacent Eu^3+^ ions. To determine the specific type of multipolar interaction responsible for the energy transfer, the relationship between emission intensity and activator concentration was further analyzed using the Van Uitert–Ozawa model:^19^6
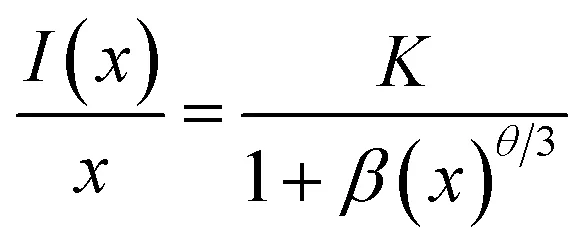
where *I*(*x*) is the luminescence intensity, *x* is the doping concentration; *θ* is a parameter associated with the interaction mechanism. Theoretical values of *θ* equal to 6, 8, and 10 correspond to dipole–dipole, dipole–quadrupole, and quadrupole–quadrupole interactions, respectively. Using the experimental luminescence intensities in the concentration quenching region, the fitting parameter was estimated to be *θ* ∼4.3. Although this value deviates noticeably from the ideal theoretical integers (*θ* = 6, 8, 10), it lies closest to *θ* = 6, which formally represents the dipole–dipole (d–d) interaction.

Rather than purely arising from experimental uncertainties or the restricted concentration range, this deviation carries a profound physical origin linked to the nature of the active coupling channels. Standard multipolar fitting models, such as the Van Uitert equation, are fundamentally constructed on the assumption of electric multipolar interactions. However, in Eu^3+^-doped systems, the deactivation of the ^5^D_1_ level (which directly feeds the emitting ^5^D_0_ state) has been demonstrated to strongly obey a magnetic-dipole (MD) coupling mechanism rather than a pure electric-dipole one.^37^ The co-existence and interference of this MD coupling pathway alongside the electric d–d interactions introduce a non-negligible perturbation to the relaxation kinetics, leading to the observed shift in the fitted *θ* value.

Nonetheless, supported by literature on structurally analogous rare-earth-doped hosts, the concentration quenching observed above 5 mol% Eu^3+^ can still be primarily attributed to non-radiative energy migration among neighboring Eu^3+^ ions, with the electric dipole–dipole coupling playing the dominant role in the long-range multipolar interactions.

#### 3.2.6. Luminescence decay behavior

**Fig. 14:**
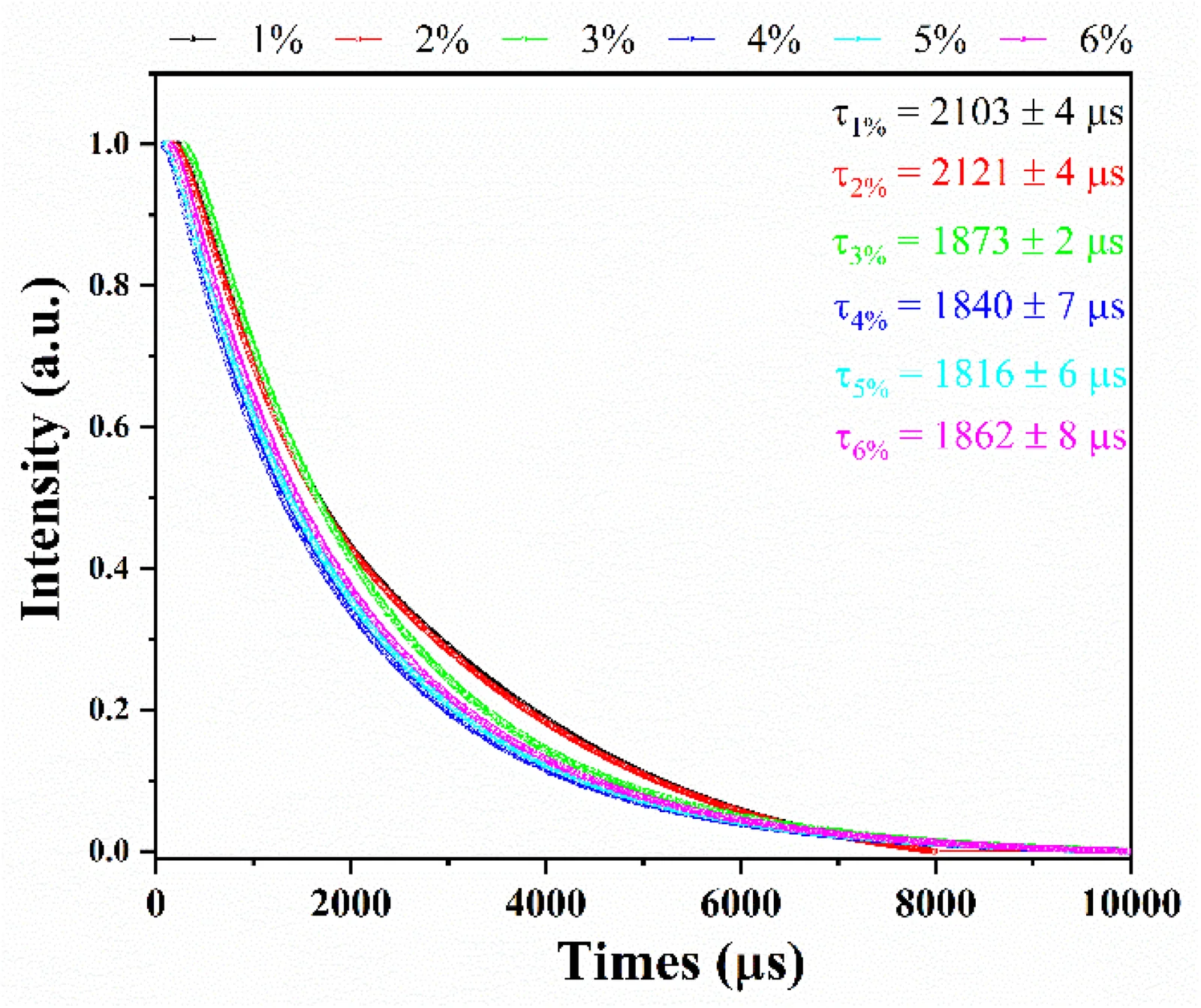
Time-resolved fluorescence spectra of Eu-doped SrY_2_O_4_ samples at concentrations from 1–6% corresponding to excitation wavelengths of 254 nm and emission wavelengths of 616 nm.

To further elucidate the influence of Eu^3+^ concentration on the luminescence dynamics of the SrY_2_O_4_ host, time-resolved photoluminescence measurements were performed for SrY_2_O_4_:*x*% Eu^3+^ (*x* = 1–6 mol%) phosphors. The decay curves were recorded by monitoring the 616 nm emission (^5^D_0_ → ^7^F_2_ transition) under 254 nm excitation and are presented in Fig. 14. The analysis results show that, at low concentrations of 1–3%, the experimental attenuation curves can be well fitted by a simple exponential function, whereas samples with concentrations of 4–6% fit well to a double exponential function in eqn (7). This result is attributed to the fact that at low concentrations, the distance between Eu^3+^ ions is large. Hence, energy transfer between them occurs with lower probability, and the emission process mainly involves transitions from the excited ^5^D_0_ state to the ^7^F_J_ states. Meanwhile, at high concentrations, non-radiative energy transfer occurs between Eu^3+^ ions.7
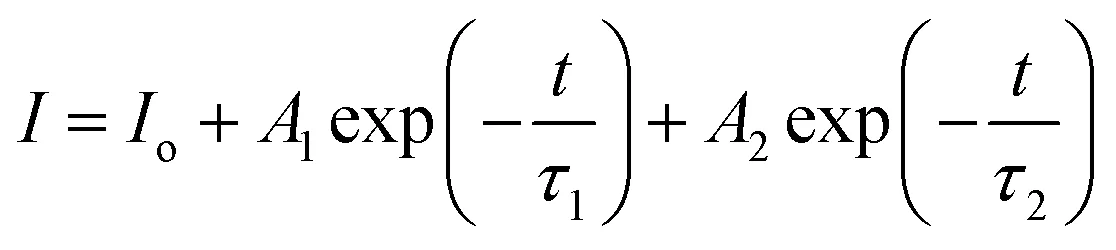
where *I*(*t*) is the fluorescence intensity at time *t*, *A*_1_ and *A*_2_ are fitting constants, and *τ*_1_ and *τ*_2_ represent the fast and slow decay components, respectively. The average lifetime *τ*_exp_ was calculated using the following expression:8
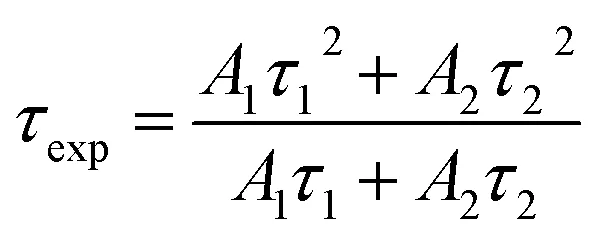


The calculated decay parameters and average lifetimes are summarized in Table 3. The SrY_2_O_4_:1% Eu^3+^ sample exhibits an average lifetime of approximately 2103 µs. With increasing Eu^3+^ concentration, the lifetime decreases gradually and reaches a minimum value of approximately 1816 µs for the 5 mol% Eu^3+^ sample, which corresponds to the optimum luminescence intensity.

**Table 3:** Decay fitting parameters and average lifetimes (exp) of SrY_2_O_4_:*x*% Eu^3+^ (*x* = 1–6) phosphors

Eu%	*A*_1_	*τ*_1_ (µs)	*A*_2_	*τ*_2_ (µs)	*τ*_exp_ (µs)	*στ*_exp_ (µs)	*R* ^2^ %
1	1.1482	2103 ± 4	0	0	2103	± 4	**99.7**
2	1.1160	2121 ± 4	0	0	2121	± 4	**99.8**
3	1.2117	1873 ± 2	0	0	1873	± 2	**99.9**
4	0.0716	721 ± 41	1.0238	1872 ± 4	1840	± 7	**99.9**
5	0.1052	651 ± 20	0.9782	1866 ± 3	1816	± 6	**99.9**
6	0.0388	758 ± 95	1.0757	1879 ± 5	1862	± 8	**99.9**

A slight increase in lifetime is observed when the Eu^3+^ concentration is further increased to 6 mol%. The gradual reduction in lifetime with increasing Eu^3+^ concentration can be attributed to the decreasing average distance between neighboring activator ions. As the Eu^3+^ concentration increases, energy migration among Eu^3+^ centers become more efficient, enhancing the probability of non-radiative energy transfer processes. Consequently, the effective lifetime of the ^5^D_0_ excited state decreases. Similar behavior has been widely reported for rare-earth-doped oxide phosphors approaching the concentration-quenching regime. It is noteworthy that the variation in lifetime is relatively small over the investigated concentration range, indicating that the SrY_2_O_4_ host effectively suppresses non-radiative losses even at elevated Eu^3+^ concentrations. This observation is consistent with the concentration-quenching analysis discussed above, where the optimum emission intensity is obtained at 5 mol% Eu^3+^ and the subsequent decrease in luminescence intensity remains moderate. The combined analysis of the decay behavior, concentration-dependent emission intensity, and critical-distance calculations suggests that the luminescence dynamics of the SrY_2_O_4_:*x*% Eu^3+^ phosphors are governed primarily by long-range energy transfer among Eu^3+^ ions. As the activator concentration increases, enhanced multipolar interactions promote energy migration between neighboring Eu^3+^ centers, leading to a gradual shortening of the excited-state lifetime and ultimately contributing to concentration quenching at high dopant concentrations.

#### 3.2.7. Chromaticity coordinates and color purity

The chromaticity coordinates and color purity of the SrY_2_O_4_:*x*% Eu^3+^ phosphors were evaluated to assess their suitability for red-light-emitting applications. The color purity (CP) was calculated from the Commission Internationale de l'Éclairage (CIE 1931) chromaticity coordinates using the following equation:^38^9
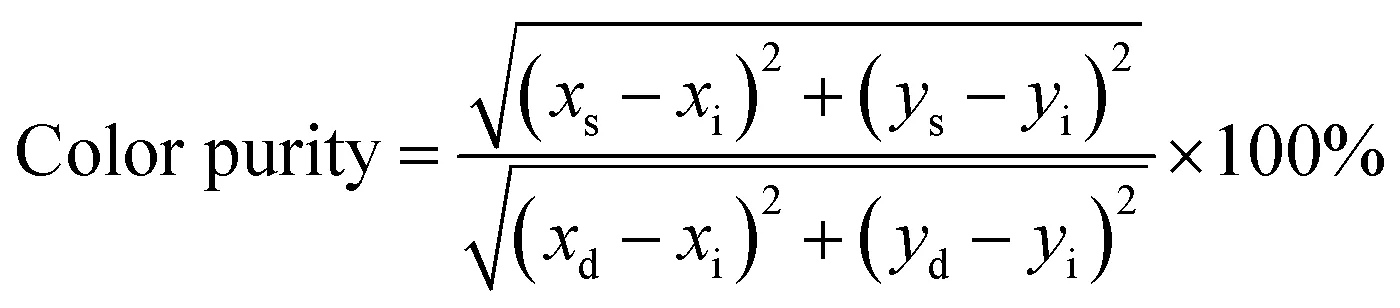
where (*x*_s_, *y*_s_) are the chromaticity coordinates of the phosphor sample, (*x*_i_, *y*_i_) are the coordinates of the standard white point (*x*_i_ = 0.3101, *y*_i_ = 0.3162), and (*x*_d_, *y*_d_) are the chromaticity coordinates corresponding to the dominant monochromatic wavelength on the CIE boundary.

**Fig. 15:**
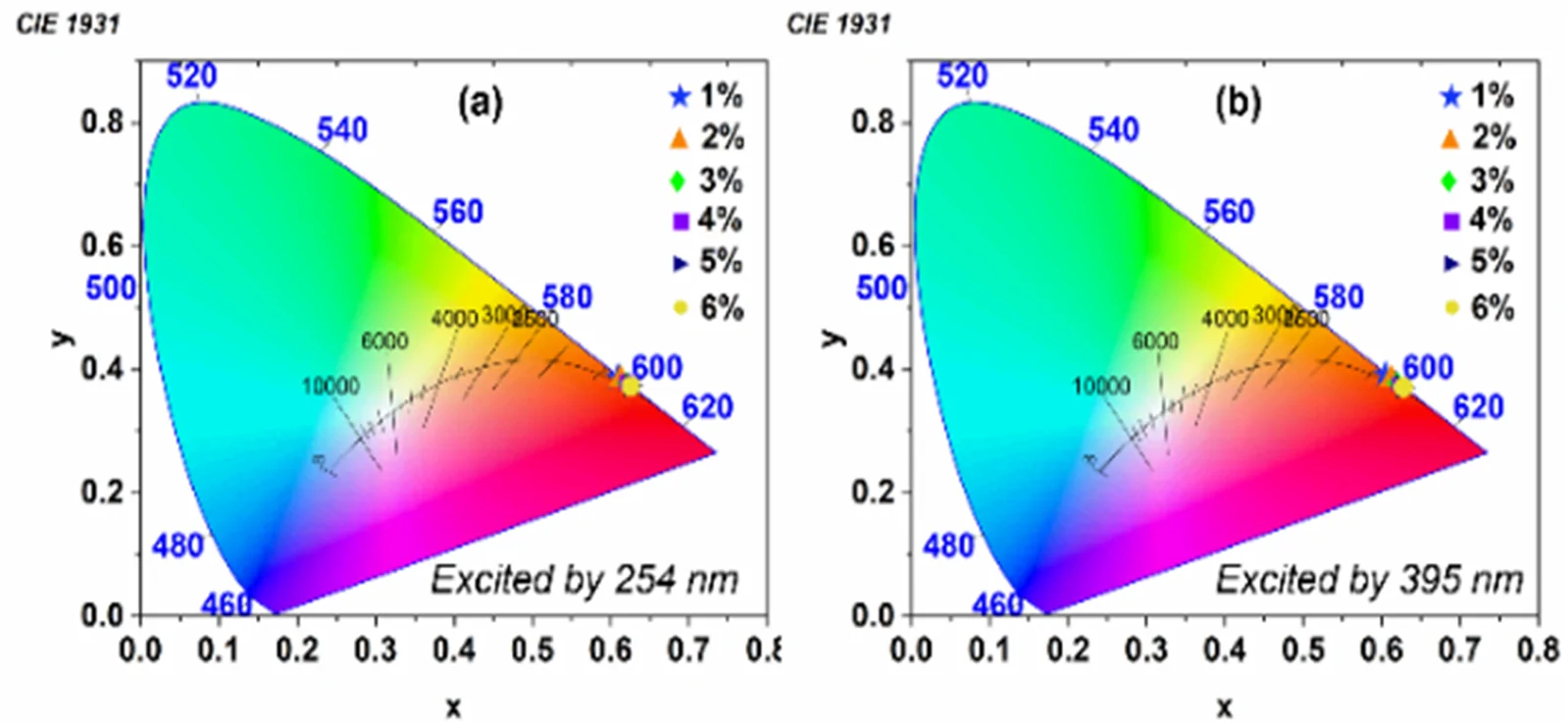
CIE 1931 chromaticity diagrams of SrY_2_O_4_:*x*% Eu^3+^ (*x* = 1–6 mol%) phosphors under excitation at (a) 254 nm and (b) 395 nm. The chromaticity coordinates of all samples are located in the red region of the CIE diagram and shift slightly toward deeper red emission with increasing Eu^3+^ concentration.

The calculated chromaticity coordinates and corresponding color purities are summarized in Table 4. All samples are located in the red region of the CIE diagram, confirming that the luminescence is dominated by the characteristic Eu^3+^ emission. The observed chromaticity originates primarily from the intense ^5^D_0_ → ^7^F_2_ forced-electric dipole transition centered at approximately 611 nm, which contributes the majority of the emitted light in the red spectral region. The calculated color purity increases with Eu^3+^ concentration and reaches values as high as 89.7% for samples containing 4–6 mol% Eu^3+^. Such high color purity indicates excellent spectral monochromaticity and demonstrates that the emission is concentrated within a narrow red wavelength range. The high-purity red emission is consistent with the dominance of the hypersensitive ^5^D_0_ → ^7^F_2_ transition and the relatively weak contribution of other Eu^3+^ emission bands. The excellent chromaticity characteristics, together with the strong red emission intensity and good thermal stability, demonstrate that SrY_2_O_4_:*x*% Eu^3+^ phosphors are promising candidates for red-emitting components in phosphor-converted white light-emitting diodes (pc-WLEDs), display technologies, and other solid-state lighting applications requiring high color saturation. The analysis results showed that the Eu^3+^ ion-doped SrY_2_O_4_ fluorescent material emitted red light with high color purity, up to 89.7% for samples with Eu^3+^ doping concentrations from 4–6%. The CIE 1931 chromaticity coordinates of SrY_2_O_4_:*x*% Eu^3+^ (*x* = 1–6 mol%) phosphors under 254 and 395 nm excitation are presented in Fig. 15. Under both excitation wavelengths, all samples exhibit chromaticity coordinates located in the red region of the CIE diagram, confirming the dominant red emission originating from the ^5^D_0_ → ^7^F_2_ transition. As the Eu^3+^ concentration increases from 1 to 6 mol%, the emission coordinates gradually shift toward the red corner of the chromaticity diagram, indicating an enhancement of color saturation and red purity. The chromaticity coordinates obtained under 254 and 395 nm excitation are very similar, suggesting that the emission color is largely independent of the excitation wavelength. This behavior indicates that the radiative recombination process is dominated by the intrinsic 4f–4f transitions of Eu^3+^ ions, which are only weakly affected by the excitation pathway. The optimized SrY_2_O_4_:5% Eu^3+^ sample exhibits coordinate close to the edge of the red region, corresponding to a high color purity of approximately 85.9%, which is favorable for red phosphor applications in phosphor-converted light-emitting devices and display technologies.

**Table 4:** Color coordinates, color temperature, *I*_534_/*I*_611_ ratio, *Ω*_2,4_ parameters, theoretical lifetime, experimental lifetime, and internal quantum efficiency of the SrY_2_O_4_:Eu^3+^ material

Samples	Chromaticity diagram (*x*, *y*)		CCT (K)	*I*_534_/*I*_611_ (%)	*Ω*_2_ (×10^−20^ cm^2^)	*Ω*_4_ (×10^−20^ cm^2^)	*R*	Color purity (%)	*A*_rad_ (s^−1^)	*τ*_exp_ (µs)	*η* (%)
SrY_2_O_4_:1% Eu^3+^	0.604	0.392	1401	8.04	2.623	0.699	1.770	82.44	356.25	2103	74.92
SrY_2_O_4_:2% Eu^3+^	0.612	0.385	1333	6.74	2.716	0.807	1.823	83.30	361.01	2121	76.57
SrY_2_O_4_:3% Eu^3+^	0.620	0.378	1266	4.70	2.748	0.688	1.854	84.80	366.03	1873	68.57
SrY_2_O_4_:4% Eu^3+^	0.623	0.376	1246	4.04	2.753	0.777	1.886	84.96	366.03	1840	67.35
SrY_2_O_4_:5% Eu^3+^	0.627	0.372	1213	3.07	2.795	0.771	1.857	85.86	372.99	1816	67.74
SrY_2_O_4_:6% Eu^3+^	0.628	0.371	1206	3.06	2.733	0.709	1.844	85.81	367.24	1862	68.38

### 3.3. Judd–Ofelt parameter analysis

To obtain further insight into the local crystal-field environment surrounding Eu^3+^ ions and its evolution with dopant concentration, the photoluminescence spectra were analyzed using Judd–Ofelt (J–O) theory. Since the forced-electric dipole transitions of Eu^3+^ are highly sensitive to the local symmetry and bonding environment, the J–O intensity parameters provide valuable information regarding the structural characteristics of the luminescent centers.^39^ For Eu^3+^ ions, the transitions from the ^5^D_0_ excited state to the ^7^F_2_, ^7^F_4_, and ^7^F_6_ levels are forced-electric dipole transitions, whereas the ^5^D_0_ → ^7^F_1_ transition is a magnetic-dipole transition whose intensity is only weakly affected by the local crystal field. Therefore, the magnetic-dipole transition is commonly used as an internal reference for determining the J–O intensity parameters. The relationship between the integrated emission intensities and the J–O parameters *Ω*_2_, *Ω*_4_, and *Ω*_6_ is given by:^20,39^10
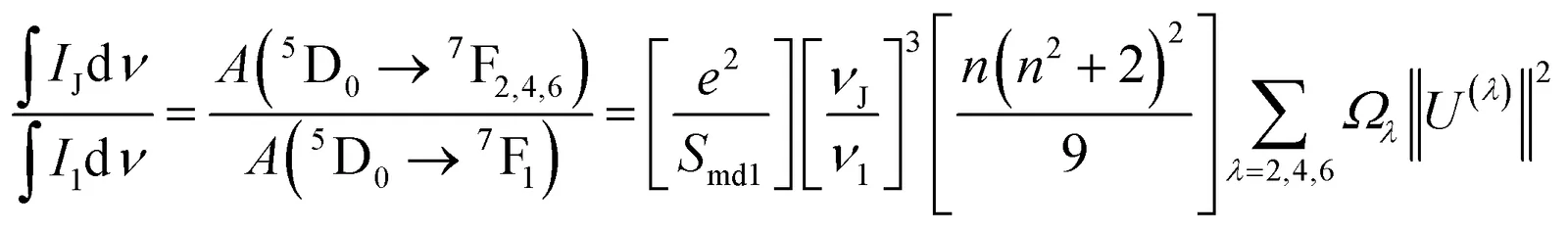


Because no measurable ^5^D_0_ → ^7^F_6_ emission was observed in the investigated spectral range, only *Ω*_2_ and *Ω*_4_ were evaluated. For the ^5^D_0_ → ^7^F_2_ transition, the reduced matrix elements are (‖*U*^(2)^‖ = 0.0032) and (‖*U*^(4)^‖ = ‖*U*^(6)^‖ = 0), whereas for the ^5^D_0_ → ^7^F_4_ transition, (‖*U*^(4)^‖ = 0.0023) and (‖*U*^(2)^‖ = 0), the refractive index values of SrY_2_O_4_ used for the respective wavelength ranges were *n* = 2.02 for the 584–603 nm range (^5^D_0_ → ^7^F_1_), *n* = 2.01 for the 604–666 nm range (^5^D_0_ → ^7^F_2_), and *n* = 1.99 for the 682–720 nm range (^5^D_0_ → ^7^F_4_). The calculated Judd–Ofelt (J–O) intensity parameters are summarized in Table 2. As the Eu^3+^ concentration increases from 1 to 5 mol%, the *Ω*_2_ parameter varies slightly from 2.623 × 10^−20^ cm^2^ to 2.79510^−20^ cm^2^. Contrary to systems with highly distorted coordination spheres, these obtained *Ω*_2_ values are relatively low, which strongly indicates that although the Eu^3+^ ions occupy the non-centrosymmetric 4c Wyckoff sites (site symmetry *C*_s_), the local environment exhibits a marginal degree of angular distortion and structurally approaches a quasi-centrosymmetric configuration.^40^ The minor, gradual increase in *Ω*_2_ with doping concentration suggests a slight, progressive enhancement of the local crystal-field distortion as more Y^3+^ sites are substituted by the slightly larger Eu^3+^ ions. This structural evolution is in excellent agreement with the lattice expansion verified by XRD and the progressive broadening of Raman bands, both pointing to a subtle increase in local structural disorder.

On the other hand, the *Ω*_4_ parameter remains nearly stable, maintaining a low average value of approximately 0.7510^−20^ cm^2^. In accordance with Judd–Ofelt physical principles, *Ω*_4_ serves as a more reliable indicator of the Eu–O bond covalency because it is fundamentally governed by the metal–ligand bond length and is largely insensitive to localized angular distortions,^41,42^ unlike *Ω*_2_ which is heavily perturbed by ligand polarizability and angular deviations. The highly restricted variation in *Ω*_4_ indicates that Eu^3+^ incorporation induces minimal alterations to the host's bond covalency and long-range structural features. This observation is consistent with the XRD and Raman analyses, confirming that the overall orthorhombic SrY_2_O_4_ framework is structurally well-preserved across the investigated concentration range.

It is also worth noting that the experimental and computational determination of the Judd–Ofelt intensity parameters (*Ω*_*λ*_) typically carries an inherent uncertainty margin of approximately 10–15%, arising from factors such as spectral integration, lifetime measurement limits, and theoretical approximations of the JO model.^43^ Given this margin, the slight variations observed in both *Ω*_2_ (2.623 × 10^−20^ cm^2^ to 2.795 × 10^−20^ cm^2^) and the nearly flat trend of *Ω*_4_ (averaging ∼0.75 × 10^−20^ cm^2^) are statistically within the experimental error limits.

Discussing these variations within the context of their error margins is crucial, as it prevents the overinterpretation of minor numerical fluctuations. The fact that both parameters remain fundamentally constant within the error limits strongly supports the conclusion that the local coordination polyhedra around the Eu^3+^ active centers (the 4c site with *C*_s_ symmetry) maintain highly stable local symmetry, bond lengths, and covalent character, without undergoing any abrupt or severe structural phase transitions across the investigated doping concentration range.

The radiative transition probability A(*J*, *J*′) for each transition was calculated using:^44^11
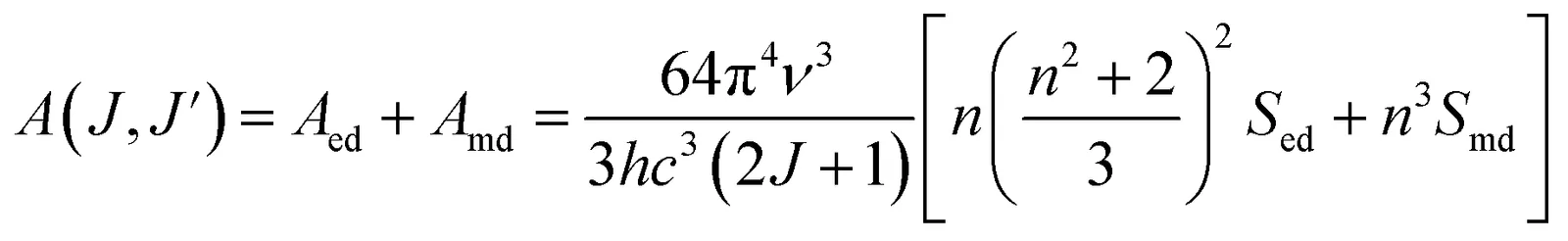


The total radiative transition probability *A*_rad_ of the excited ^5^D_0_ state to all lowe ^7^F_J_ states is determined by summing the individual transition probabilities *A*_*JJ*′_:^45^12
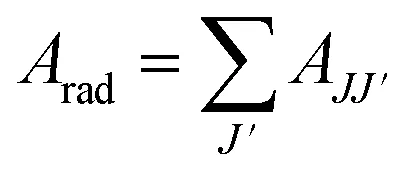


The theoretical branching ratio *β*_cal_ for each emission transition was obtained from :^46^13
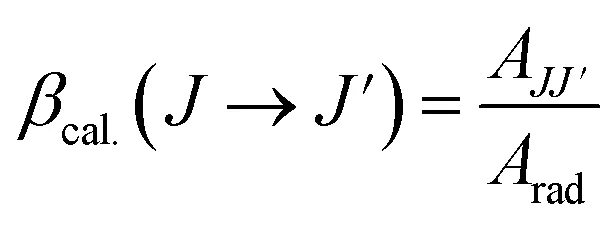
he derived internal quantum efficiency *η* could be determined using the experimental decay time *τ*_exp_ and the calculated total radiative transition rate *A*_rad_:^47^

14 *η* = *A*_rad_·*τ*_exp_

The calculated optical parameters are listed in Table 4. The total radiative transition probability, Arad, increases slightly from 365.25 s^−1^ for the 1 mol% Eu^3+^ sample to a maximum of 372.99 s^−1^ for the 5 mol% Eu^3+^ sample, then decreases slightly at higher concentrations. This behavior is consistent with the concentration-dependent evolution of luminescence intensity. The increase in the radiative transition rate near the optimum concentration reflects an enhancement in the transition probability associated with the dominant ^5^D_0_–^7^F_2_ emission. When the Eu^3+^ concentration exceeds 5 mol%, concentration quenching induced by non-radiative energy migration among neighboring Eu^3+^ ions becomes significant, resulting in reduced emission intensity. The experimental lifetimes remain close to 1816–2121 ms across the investigated concentration range, indicating that the SrY_2_O_4_ host maintains a relatively low density of non-radiative defects even at elevated Eu^3+^ concentrations. Consequently, the calculated internal quantum efficiency remains nearly constant at approximately 67%, while the color purity exceeds 82% for all compositions. These results demonstrate that the local crystal environment of Eu^3+^ ions is preserved over a broad concentration range, enabling stable red emission with high color saturation and good luminescence efficiency. The combination of relatively large *Ω*_2_ values, high color purity, long luminescence lifetime, and stable quantum efficiency confirms that SrY_2_O_4_ provides a favorable host lattice for Eu^3+^ luminescent centers. These characteristics, together with the strong red emission under near-ultraviolet and blue-light excitation, highlight the potential of SrY_2_O_4_:*x*% Eu^3+^ phosphors for solid-state lighting applications.

Compared with previously reported Eu^3+^-activated oxide phosphors, SrY_2_O_4_:Eu^3+^ exhibits competitive thermal stability (*E*_a_ = 0.258 eV), relatively high internal quantum efficiency (∼68%), and excellent color purity (∼86%). Although its color purity is lower than that of some molybdate- and vanadate-based hosts, the material offers the advantages of a chemically stable oxide framework, broad UV excitation capability, and efficient red emission under both 254 and 395 nm excitation.

It is worth noting that the calculated quantum efficiencies (*η*) of the prepared SrY_2_O_4_:Eu^3+^ phosphors, while exhibiting a high values peaking at 2 mol%, are lower than those reported for similar systems in the literature, such.^48^ This discrepancy can be physically understood by analyzing the localized non-radiative deactivation pathways.

As revealed by our FTIR spectra, a discernible absorption band associated with the stretching vibrations of hydroxyl (OH^−^) groups is present at approximately 3400–3600 cm^−1^. The OH^−^ oscillators possess exceptionally high vibrational energy (∼3400 cm^−1^) compared to the intrinsic phonon energy of the host lattice (500–600 cm^−1^). Since the energy gap between the emitting ^5^D_0_ level and the lower-lying ^7^F_6_ state of Eu^3+^ is relatively small (12 000 cm^−1^), only about 3–4 vibrational phonons of the OH^−^ groups are required to bridge this energy gap.

Consequently, the presence of even a trace amount of surface-adsorbed OH- impurities activates an extremely dominant and rapid non-radiative multi-phonon relaxation (MPR) pathway. This inter-molecular energy dissipation bypasses the radiative channels, significantly shortening the experimental lifetime (*τ*_exp_) and leading to the observed lower quantum efficiencies compared to highly optimized or fully anhydrous samples reported in ref. 48. This correlation highlights that minimizing external hydroxyl contaminants during synthesis remains a key prerequisite for maximizing the absolute quantum yield of these red-emitting phosphors.

### 3.4. Thermal stability of materials

**Fig. 16:**
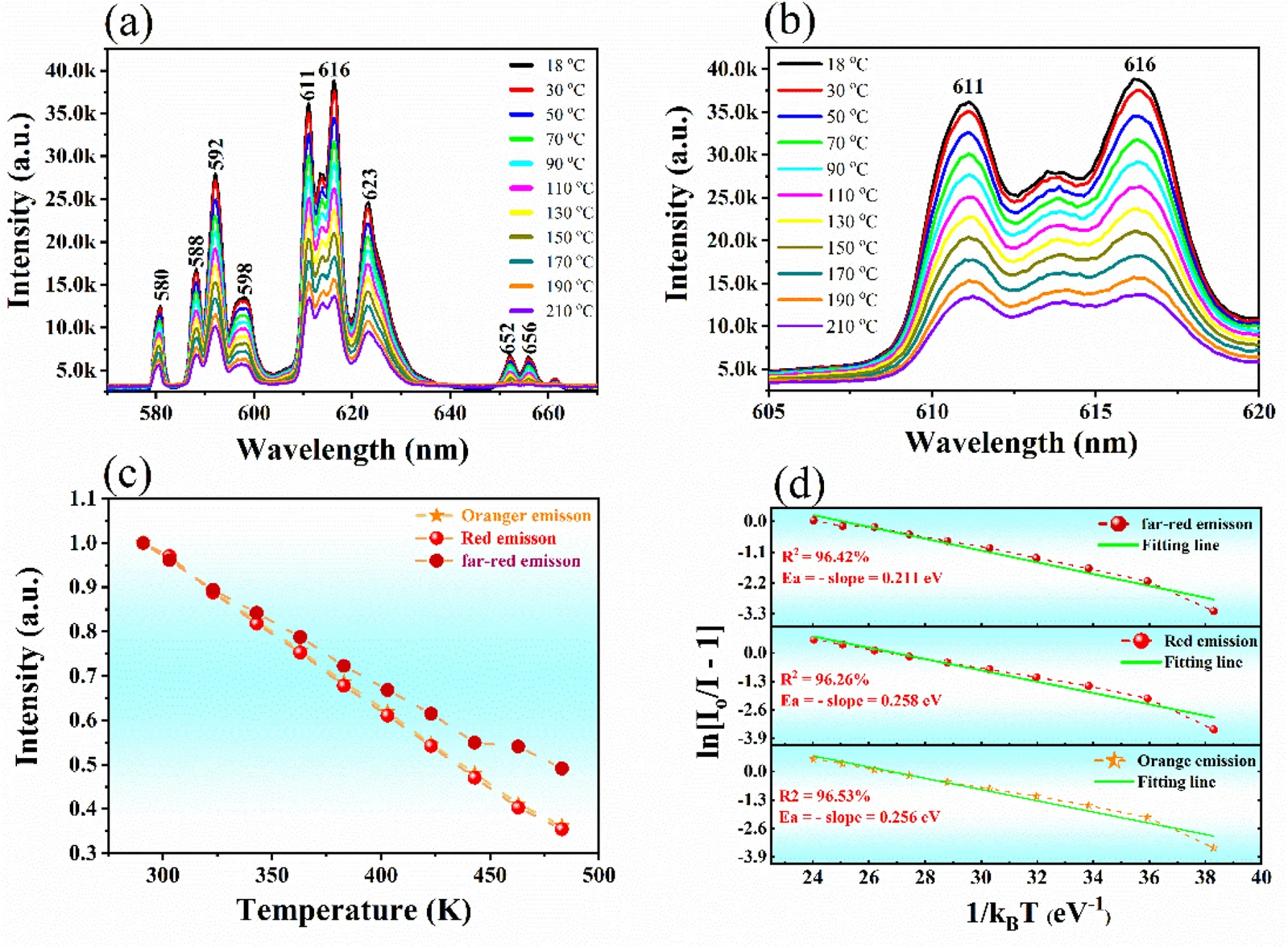
Temperature-dependent photoluminescence properties of SrY_2_O_4_:5% Eu^3+^ phosphor under 395 nm excitation: (a and b) emission spectra recorded from room temperature to 210 °C; (c) normalized emission intensities of the orange ^5^D_0_ → ^7^F_1_), red (^5^D_0_ → ^7^F_2_), and far-red (^5^D_0_ → ^7^F_4_) transitions as a function of temperature; and (d) Arrhenius plots of ln[(*I*_0_/*I*) − 1] *versus* 1/*k*_B_*T* used for determining the thermal activation energy.

Thermal stability is a critical parameter for evaluating the suitability of phosphors in high-power LED applications because the operating temperature of LED devices can significantly affect luminescence performance. To assess the thermal stability of the synthesized phosphors, temperature-dependent photoluminescence measurements were performed on the optimized SrY_2_O_4_:*x*% Eu^3+^ sample under 395 nm excitation over the temperature range from room temperature to 210 °C, as shown in Fig. 16.

The emission spectra show that the characteristic Eu^3+^ emission bands maintain their spectral positions throughout the investigated temperature range, indicating that the local crystal-field environment around the Eu^3+^ ions remains stable upon heating. However, the emission intensity gradually decreases with increasing temperature. This thermal quenching behavior is attributed to the enhancement of lattice vibrations at elevated temperatures, which increases the probability of non-radiative relaxation through multiphonon-assisted processes.

Consequently, a larger fraction of the excitation energy is dissipated through phonon interactions rather than radiative transitions from the ^5^D_0_ excited state. To quantitatively evaluate the thermal-quenching process, the activation energy *E*_a_ was determined using the Arrhenius equation:^7,49^16
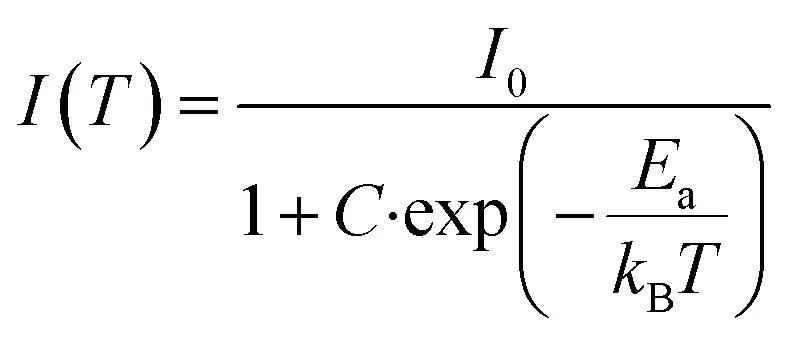
where *I*(*T*) and *I*_0_ represent the emission intensities at temperature (*T*) and room temperature, respectively, *k*_B_ = 8.6173 × 10^−5^ eV K^−1^ is the Boltzmann constant, and *E*_a_ is the thermal activation energy associated with non-radiative relaxation.

Eqn (16) can be rearranged into the linear form:17
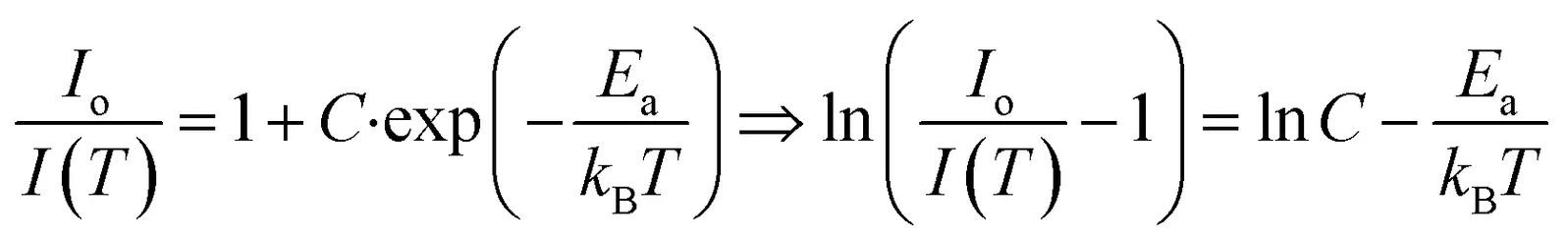


The activation energies (*E*_a_) derived from the Arrhenius plots for the orange, red, and far-red emission bands were determined to be 0.211, 0.258, and 0.256 eV, respectively. The minor variations in *E*_a_ across these bands, all originating from the same ^5^D_0_ excited state, stem from peak overlap induced by Stark splitting. To ensure consistency, the analysis was focused on the most prominent peak within each emission band, yielding an average activation energy of approximately 0.242 eV. This substantial energy barrier indicates high thermal stability against the non-radiative quenching process. Such an activation energy is comparable to or higher than that reported for many Eu^3+^-activated oxide phosphors, demonstrating the favorable thermal stability of the SrY_2_O_4_ host. A comparison of the thermal-quenching activation energy and luminescence performance of SrY_2_O_4_:5% Eu^3+^ with several representative Eu^3+^-activated phosphors reported in the literature is presented in Table 5. The obtained activation energy is comparable to those reported for K_3_Mg_4_LiSi_12_O_30_:Eu^3+^ (0.249 eV),^53^ LiSrVO_4_:Eu^3+^ (0.28 eV),^54^ and Ba_3_Lu_4_O_9_:Eu^3+^ (0.210 eV),^58^ indicating competitive thermal stability.

**Table 5:** Comparison of research results with several other Eu^3+^ ion-doped fluorescent material systems

Phosphors	*λ*_ex_ (nm)	*λ*_em_ (nm)	Color purity (%)	*η* (%)	*E*_a_ (eV)	Ref.
Ba_2_GdVO_6_:Eu^3+^	395, 466	611	99.17	96.67	0.190	50
Ca_2_GdTaO_6_:Eu^3+^	465	613	97.19	91.43	0.20	51
Y_2_(MoO_4_)_3_:Eu^3+^	395	618	98.17	—	0.31	52
K_3_Mg_4_LiSi_12_O_3_:Eu^3+^	396	615	99.0	16.03	0.249	53
LiSrVO_4_:Eu^3+^	396, 466	617	96.4	—	0.28	54
SrTa_2_O_6_:Eu^3+^	465	618	—	36.45	—	55
GdSr_2_AlO_5_:Eu^3+^	268	612	98.6	67.62	0.216	56
CaTiO_3_:Eu^3+^	398, 466	613	85	—	—	57
Ba_3_Lu_4_O_9_:Eu^3+^	396	614	98	82	0.210	58
SrY_2_O_4_:Eu^3+^	254	611	—	95.42	—	15
SrY_2_O_4_:5% Eu^3+^	254, 395	611	85.86	67.66	0.258	**This work**

Furthermore, the emission intensity of SrY_2_O_4_:5% Eu^3+^ retains approximately 76% of its room-temperature value at 150 °C (423 K), demonstrating good resistance to thermal quenching under practical operating conditions. Combined with its high color purity, long luminescence lifetime, and efficient red emission under near-ultraviolet excitation, these results confirm that SrY_2_O_4_:Eu^3+^ is a promising red-emitting phosphor for warm WLED applications.

## 4. Conclusions

Eu^3+^-activated SrY_2_O_4_ red-emitting phosphors were successfully synthesized *via* a conventional solid-state reaction route at 1200 °C. Structural analyses based on XRD, Raman spectroscopy, HRTEM, and XPS confirmed the formation of a single-phase orthorhombic SrY_2_O_4_ host lattice and demonstrated that Eu^3+^ ions preferentially substitute for Y^3+^ ions at low-symmetry lattice sites without altering the crystal structure. The incorporation of Eu^3+^ induced slight lattice expansion, local structural distortion, and a gradual reduction in the optical band gap from 6.13 to 5.93 eV. The phosphors exhibited intense red emission dominated by the ^5^D_0_ → ^7^F_2_ transition of Eu^3+^ centered at 611 nm under ultraviolet and near-ultraviolet excitation. The optimum luminescence intensity was obtained at an Eu^3+^ concentration of 5 mol%, beyond which concentration quenching occurred due to non-radiative energy transfer among neighboring Eu^3+^ ions, primarily through dipole–dipole interactions. The phosphors showed high color purity of up to 85.86%, an internal quantum efficiency of 67.74%, and long luminescence lifetimes in the millisecond range. Judd–Ofelt analysis reveals that the *Ω*_2_ value varies insignificantly within the range of (2.623 × 10^−20^ – 2.795 × 10^−20^ cm^2^), indicating that Eu^3+^ ions occupy non-centrosymmetric sites characterized by local asymmetry at *C*_s_ sites within the SrY_2_O_4_ host lattice. The small variation in *Ω*_2_ and *Ω*_4_ with increasing Eu^3+^ concentration suggests that the local crystal-field environment remains largely preserved across the investigated compositional range, resulting in stable optical properties and chromaticity coordinates. The optimized SrY_2_O_4_:5% Eu^3+^ phosphor also exhibited good thermal stability, retaining approximately 76% of its room-temperature emission intensity at 150 °C and displaying an average thermal activation energy of about 0.242 eV. These results demonstrate that SrY_2_O_4_:5% Eu^3+^ combines efficient red emission, high color purity, good thermal stability, and favorable luminescence efficiency. The results indicate that SrY_2_O_4_:Eu^3+^ is a promising red-emitting phosphor candidate for near-UV-excited phosphor-converted WLEDs, although further device-level optimization and evaluation are required. This work provides a comprehensive correlation between Eu^3+^ concentration, local crystal-field evolution, Judd–Ofelt parameters, and luminescence performance in the SrY_2_O_4_ host lattice, offering valuable guidance for the design of efficient red phosphors for solid-state lighting.

## Author contributions

Vu Thi Kim Lien: methodology, investigation, data curation, conceptualization. Le Thi Phuong: methodology, investigation. Vu Van Thu: writing – review & editing, writing – original draft, methodology, investigation, data curation, conceptualization, funding acquisition. Nguyen Dac Dien: investigation, formal analysis. Le Thi Oanh: investigation, formal analysis. Do Thi Lan Chi: investigation, formal analysis. Pham Thi Lien: investigation, formal analysis. Chu Anh Tuan: formal analysis, methodology, investigation, data curation. Pham Van Tuan: methodology, investigation, data curation. Hoang Gia Chuc: investigation, data curation. Manh Trung Tran: investigation, data curation, writing – review & editing. Le Tien Ha: writing – original draft, methodology, investigation, formal analysis, data curation.

## Conflicts of interest

There are no conflicts to declare.

## Supplementary Material

RA-OLF-D6RA05000J-s001

## Data Availability

All data generated or analyzed during this study are included in this published article. Any additional inquiries regarding the dataset may be directed to the corresponding author at thuvv@dhcd.edu.vn. Supplementary information (SI) is available. See DOI: https://doi.org/10.1039/d6ra05000j.
