# Supplementary material for: Insights into the local structure and optical performance of SrY2O4:Eu3+ red phosphors through Judd–Ofelt analysis
Source: RSC Adv. 2026 Sep 1;16(42):51217–33. doi: 10.1039/d6ra05000j (PMC13531042; doi:10.1039/d6ra05000j)
Supplement: Supplementary file 1 [file RA-016-D6RA05000J-s001.pdf]

Supplementary Information (SI) for RSC Advances.
This journal is © The Royal Society of Chemistry 2026

Supporting information

Insights into the local structure and optical performance of $SrY_2O_4:Eu^{3+}$ red phosphors through Judd–Ofelt analysis

Vu Thi Kim Lien[1,2], Le Thi Phuong[3], Vu Van Thu[4,*], Nguyen Dac Dien[4], Le Thi Oanh[4], Do Thi Lan Chi[4], Pham Thi Lien[5], Chu Anh Tuan[6], Pham Van Tuan[7], Hoang Gia Chuc[8], Manh Trung Tran[8], Le Tien Ha[3]

[1]Faculty of Natural Sciences, Duy Tan University, Da Nang, 550000, Vietnam.

[2]Institute of Theoretical and Applied Research, Duy Tan University, Hanoi, 100000, Vietnam.

[3]TNU - University of Sciences, Thai Nguyen 250000, Vietnam

[4]Faculty of Occupational Safety and Health, Trade Union University, Hanoi, 10000, VietNam

[5]Institute of Materials Science, Vietnam Academy of Science and Technology, 18 Hoang Quoc Viet, Nghia Do, Hanoi, Vietnam.

[6]Viet Nam University of Traditional Medicine, Hanoi, 100000, Vietnam.

[7]School of Materials Science and Engineering, Hanoi University of Science and Technology, No. 1, Dai Co Viet, Hanoi, Vietnam

[8]Faculty of Materials Science and Engineering, Phenikaa School of Engineering, Phenikaa University, Nguyen Trac, Duong Noi, Hanoi, Vietnam

Corresponding author's email: thuvv@dhcd.edu.vn

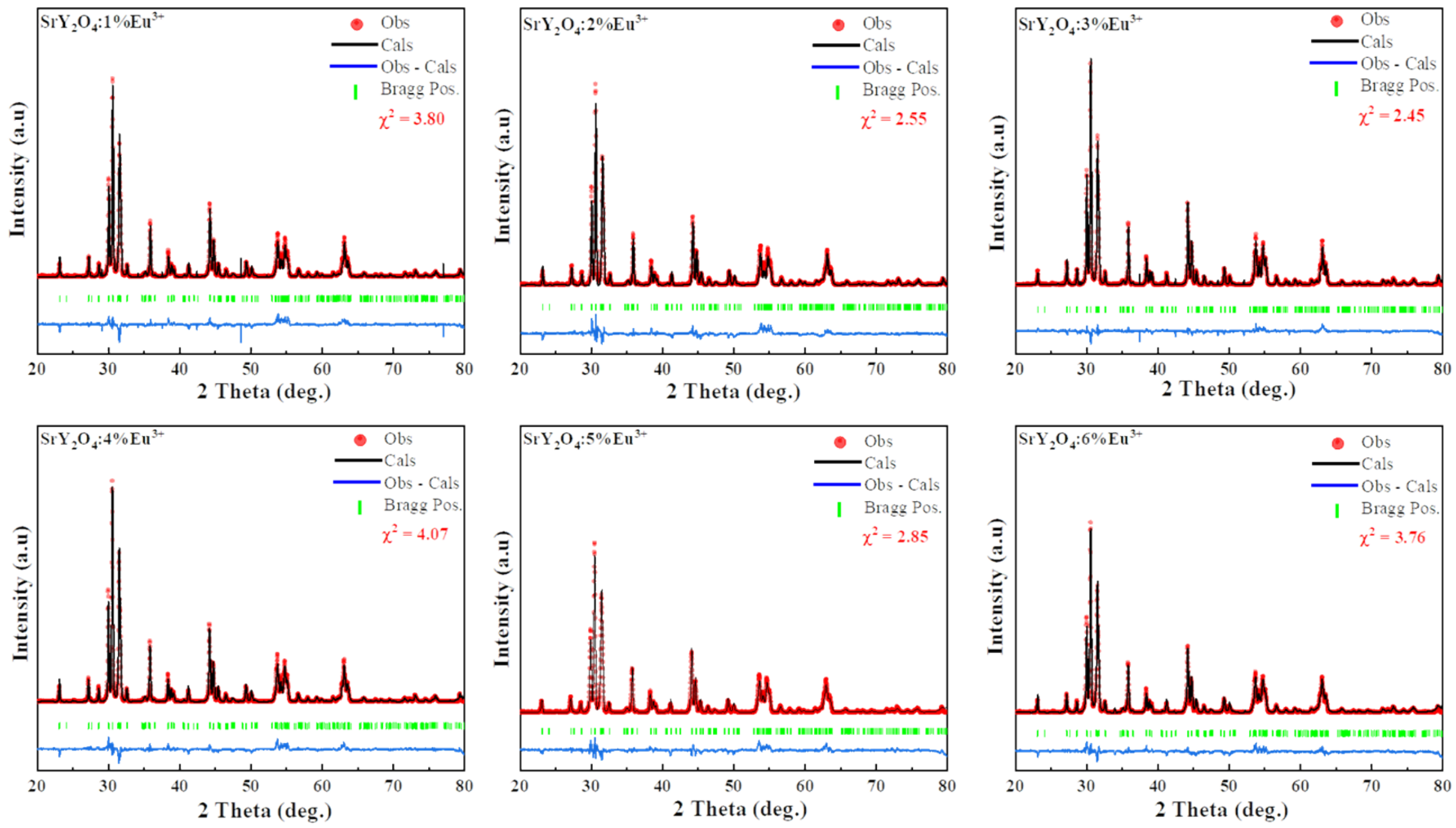

Figure S1. Rietveld refinements of SrY_2O_4:x%Eu^{3+} (x = 1-6) annealed at 1200 °C for 9h in air.

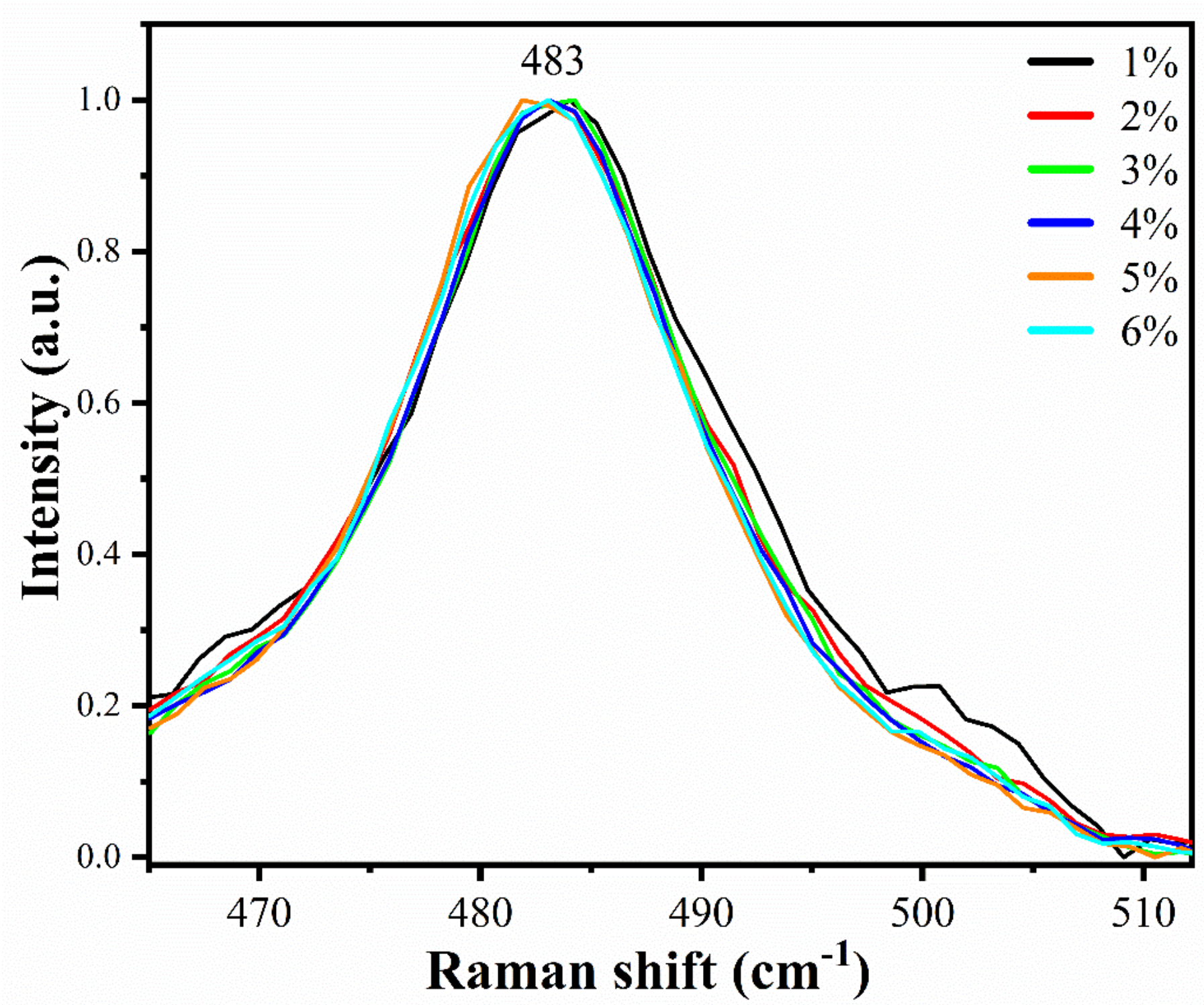

Figure S2: Normalized Raman spectra of the band centered at 483 cm^{-1} for $SrY_2O_4:Eu^{3+}$ samples with different doping concentrations (1%–6%).

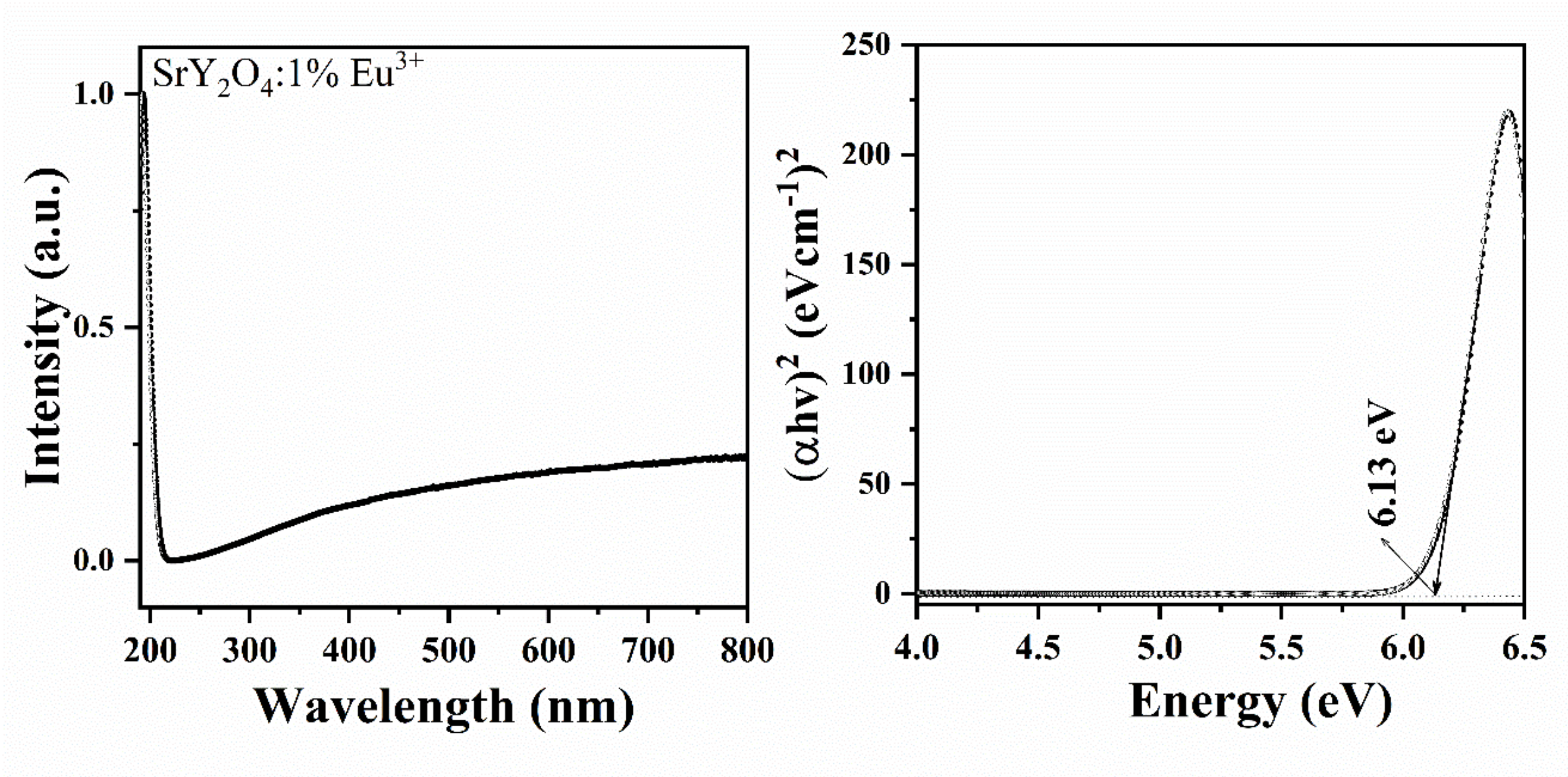

SrY2O4:1% Eu3+
Intensity (a.u.)
Wavelength (nm)
(αhν)2 (eVcm-1)2
6.13 eV
Energy (eV)

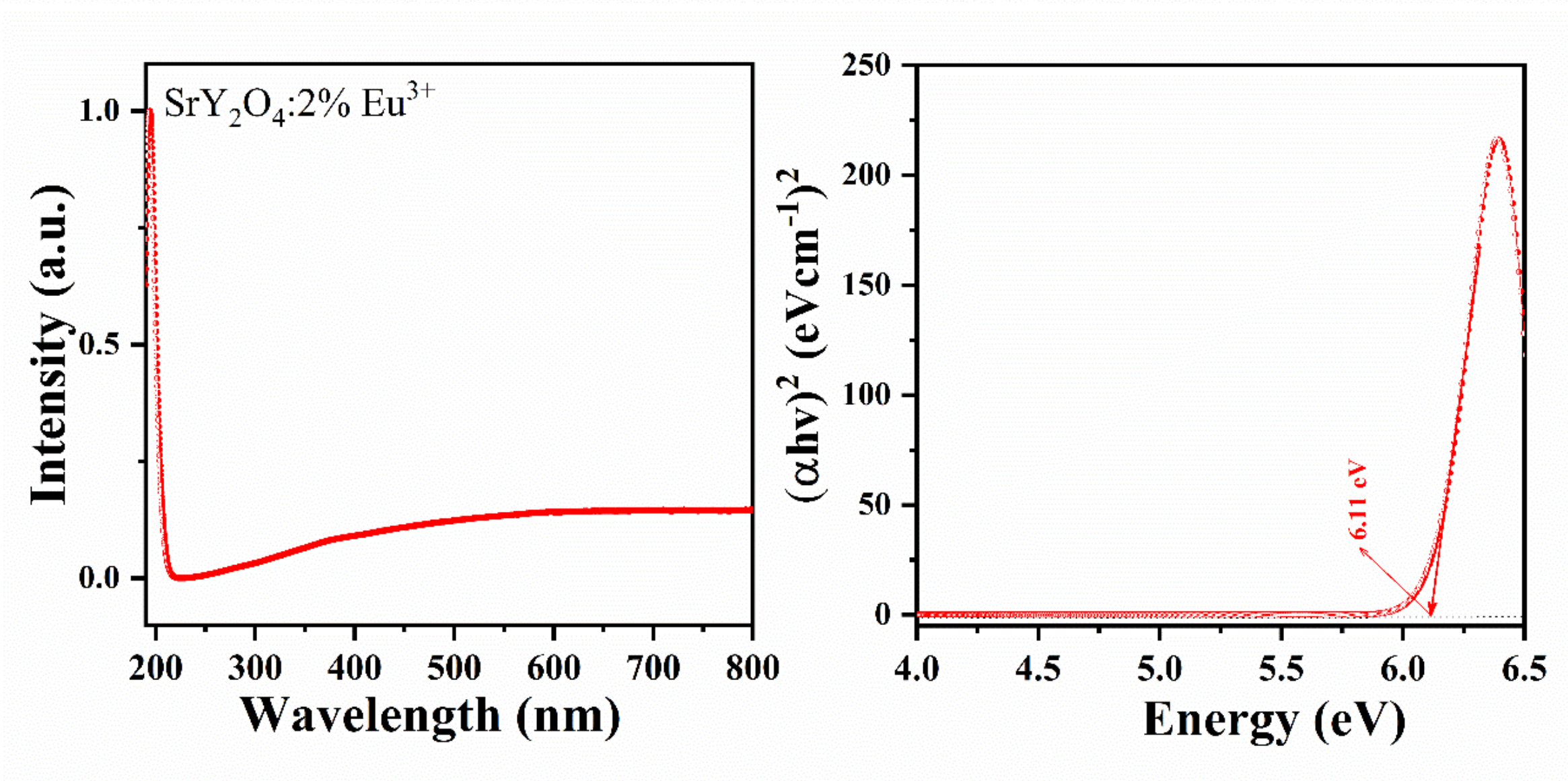

SrY2O4:2% Eu3+
Intensity (a.u.)
Wavelength (nm)
(αhν)2 (eVcm-1)2
6.11 eV
Energy (eV)

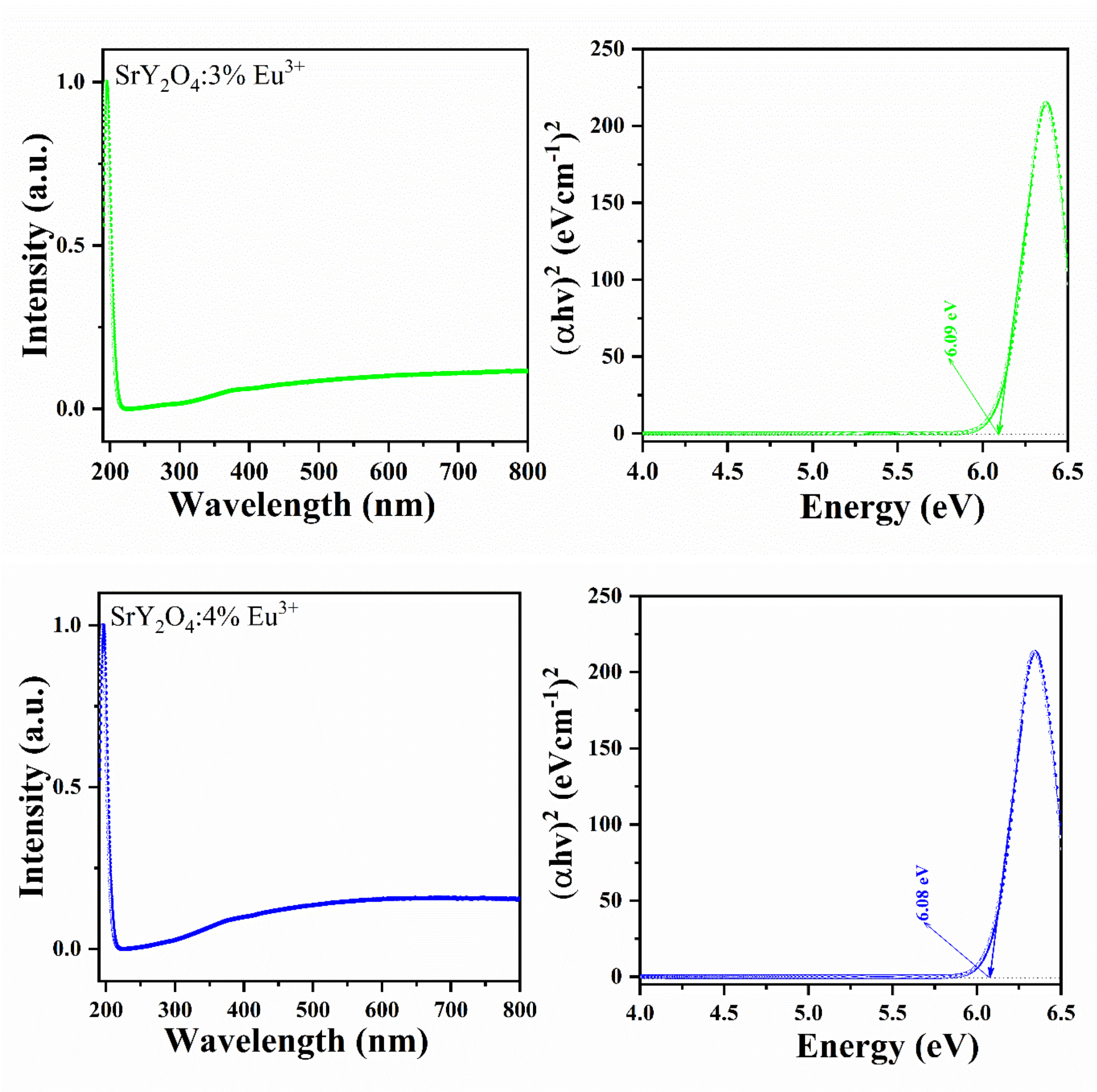

SrY2O4:3% Eu3+
Intensity (a.u.)
Wavelength (nm)
(αhv)2 (eVcm-1)2
Energy (eV)
6.09 eV
SrY2O4:4% Eu3+
Intensity (a.u.)
Wavelength (nm)
(αhv)2 (eVcm-1)2
Energy (eV)
6.08 eV

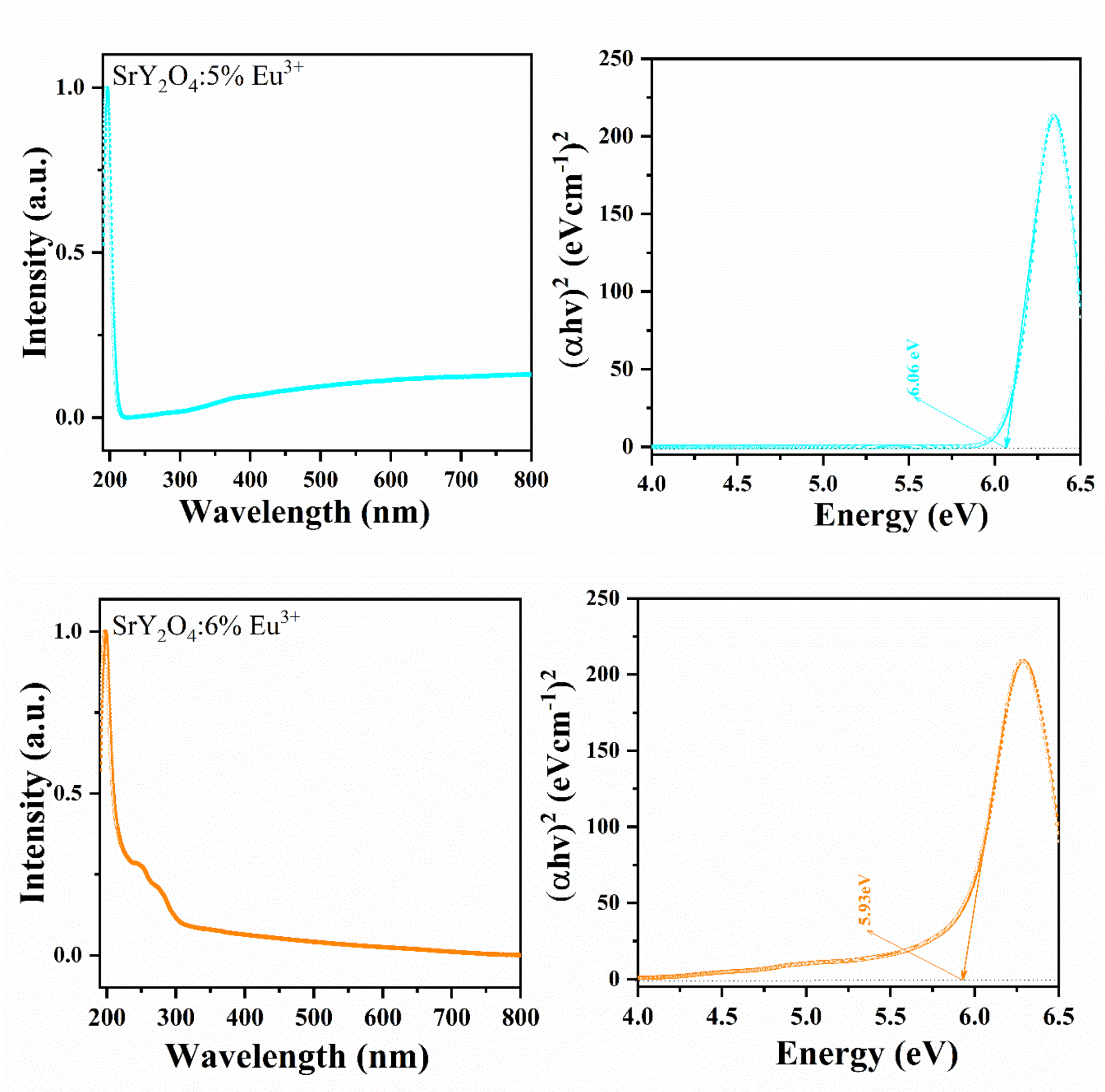

Figure S3. UV-Vis absorption spectrum and the corresponding Tauc plot [$(\alpha h\nu)^2$ vs. $h\nu$] of the SrY_2O_4:x%Eu^{3+} (x = 1-6) phosphor annealed at 1200 °C for 9 h
